# Highly Stretchable and Reliable Graphene-Based Strain Sensor for Plant Health Monitoring and Deep Learning-Assisted Crop Recognition

**DOI:** 10.34133/research.0933

**Published:** 2025-10-07

**Authors:** Yaling Wang, Pan Li, Zhizhao Liu, Jiakun Kang, Ke Liu, Yue Sun, Chunjiang Zhao, Jihua Tang, Jinpeng Cheng

**Affiliations:** ^1^ College of Science, Henan Agricultural University, Zhengzhou 450002, China.; ^2^ College of Agronomy, Henan Agricultural University, Zhengzhou 450046, China.; ^3^ National Engineering Research Center for Information Technology in Agriculture, Beijing 100097, China.

## Abstract

Stretchable sensors hold great potential for monitoring plant physiological parameters and enabling crop identification in smart agriculture. However, achieving long-term, stable, reliable monitoring of plants in dynamic environments, as well as improving crop identification accuracy, remains a substantial challenge, primarily due to the limited biocompatibility of conventional stretchable sensors. Here, we present a highly stretchable and reliable strain sensor based on a graphene/Ecoflex composite. This sensor features a mesh structure that combines graphene’s high electrical conductivity and strain sensitivity with Ecoflex’s excellent stretchability, biocompatibility, and resistance to environmental degradation. By structural optimization, the sensor achieves high sensitivity (gauge factor = 138), a low detection limit (0.1% strain), and high reliability (over 1,500 cycles), along with waterproofing and resistance to both acidic and alkaline conditions. Furthermore, the sensor conforms tightly to various plant leaves and stems without hindering growth, enabling real-time monitoring of plant growth patterns and in situ detection of mechanical damage to predict plant stress. Moreover, assisted by deep learning, it precisely classifies 8 crop types with an accuracy of 95.2%. These demonstrate that stretchable sensors based on mesh graphene/Ecoflex can operate reliably in outdoor agricultural environments even in the face of variable climatic and chemical conditions, providing a practical platform for advancing plant phenomics and smart agricultural robotics.

## Introduction

With the rapid development of global agricultural intelligence and precision, crop health monitoring and identification have become key means to improving crop yield and quality and optimizing management decisions [1–4]. Plants undergo complex and variable environmental stresses (e.g., water shortages, pest attacks, disease infections, and extreme temperatures) during their growth cycle. They often trigger subtle morphological responses, such as stem swelling, leaf curling, or surface deformation, which can be mechanically tracked using surface-mounted stretchable strain sensors [5–7]. Owing to their excellent mechanical adaptability, high stretchability, and sensitivity to small deformations, stretchable strain sensors are ideal for real-time plant growth monitoring [8–10]. However, current strain sensors, such as those based on metal nanofilms, carbon nanotubes, or polymer composites [11–13], typically suffer from poor tensile performance, limited durability, and signal degradation during prolonged use, which limits their applicability in dynamic, long-term plant monitoring scenarios. Therefore, there is an urgent need to develop a highly stretchable and reliable strain sensor for long-term and stable tracking of small deformations in plants, enabling an accurate assessment of plant health status.

Although traditional crop recognition methods, such as RGB imaging, multispectral/hyperspectral analysis, and manual feature extraction, have been widely adopted due to their high accuracy and maturity [14], they face inherent limitations in agricultural field applications. Variations in lighting, occlusion by foliage, and interference from complex backgrounds can considerably reduce recognition performance [15–17]. Moreover, high-resolution images require costly hardware and large amounts of data transmission bandwidth, which limits their application in large-scale, long-term deployment [18]. Furthermore, morphological features captured in images cannot directly reflect subtle mechanical or physiological changes in plants. In contrast, flexible strain sensors have extremely high resistance to environmental interference and require far less data storage and transmission than image data [19–21]. They can capture minute deformations related to plant physiological states and support continuous, wearable monitoring of multiple plant species [22]. Therefore, combining high-performance strain sensors with deep learning not only improves crop recognition accuracy but also overcomes the limitations of traditional image-based methods in terms of environmental sensitivity, large data volumes, and lack of physiological information, providing a scalable, cost-effective, and information-rich solution for smart agriculture.

Graphene-based materials, including graphene nanoplatelets and reduced graphene oxide, possess excellent electrical conductivity, mechanical flexibility, and high specific surface area, making them widely used in strain sensors [23]. Depending on the material configuration and sensing architecture, graphene strain sensors can operate through various mechanisms such as piezoresistive effects, interfacial tunneling resistance, and crack propagation, enabling sensitive detection of mechanical deformation in dynamic environments [24,25]. Liu et al. [26] proposed a graphene strain sensor with a gauge factor (GF) of 7.1 within a strain range of 0% to 40%. However, this sensor faces limitations in detecting small strains, and the graphene itself tends to experience a considerable decrease in conductivity during tensile deformation, which restricts its application in wearable strain sensors for plants. Constructing a mesh structure is an efficient means to enhance the mechanical performance of flexible sensors [27–29]. Yang et al. [30] developed a strain sensor using mesh structures. Although the sensor exhibited excellent stretchability (with a maximum breaking strain of 704%), its sensitivity was relatively low (GF = 32.95). Therefore, combining graphene with a mesh structure can effectively enhance its mechanical stability during the stretching process, thereby maintaining high conductivity while providing sufficient flexibility and stretchability.

In actual agricultural environments, plant surfaces frequently come into contact with chemicals such as foliar fertilizers, pesticides, and acidic/alkaline rainwater, which can substantially affect the chemical environment of the leaf or stem surface [31]. These external chemical stimuli can cause corrosion of sensing materials or electrical instability, thereby reducing their long-term reliability [32,33]. Consequently, stretchable sensors for plant health monitoring need to meet the requirements of waterproofing as well as resistance to acids and alkalis. Reliable encapsulation materials can improve the sensor’s waterproofing and resistance to acids and alkalis. Zhang et al. [34] designed an origami-inspired wearable 3-dimensional (3D) sensor to monitor plant growth in situ and online. However, their sensor module was not encapsulated, resulting in a lack of waterproofing and an inability to support long-term monitoring. Ecoflex is a silicone rubber material with excellent elasticity, high stretchability, durability, and superior biocompatibility, making it an ideal encapsulation material for highly reliable stretchable sensors [35–37]. Therefore, the sensitivity and durability of stretchable sensors can be greatly enhanced by integrating a mesh-like graphene structure with Ecoflex for encapsulation. Lv et al. [38] prepared a conductive elastomeric film (CEF) made from Ecoflex and carbon nanotubes, which had a maximum stretchable strain of 412% and the resistance changed steadily with each tensile cycle under different strains, demonstrating the excellent reliability of the Ecoflex CEF sensor. Therefore, using Ecoflex elastomer to encapsulate mesh-structured graphene-based sensors holds great potential for achieving highly reliable and sensitive plant-wearable sensors.

By attaching flexible stretchable sensors to the surface of plants, it is possible to monitor the plant’s physiological state in real time, including growth rate, humidity changes, temperature fluctuations, and stress responses to external environmental factors [39–41]. This would aid in assessing plant health, predicting pest and disease risks, and optimizing plant growth conditions. Wei et al. [42] designed a multifunctional sensor that can detect slight deformations in plants subjected to mechanical stretching or external environmental changes. This sensor provides real-time feedback on the plant’s strain state, allowing for growth and health monitoring. However, if this sensor is used for long-term plant health monitoring, its stretchability may be limited by the elasticity of the material, and it may not effectively detect external stresses. Therefore, designing a stretchable sensor with higher sensitivity, water resistance, and acid–alkali resistance properties is crucial, particularly focusing on sensor stability, long-term monitoring accuracy, and adaptability across different plant species. This will help to detect plant stress, monitor plant health, and identify plant species, ultimately enhancing agricultural intelligence. Yang et al. [43] installed cameras on robots to collect images of strawberries at different growth stages for quality and color phenotype identification. However, this method is highly susceptible to external environmental interference, and the image acquisition and recognition processes are relatively complex. As a solution, integrating stretchable sensors into robotic systems could enhance environmental interaction and data quality [44]. Lin et al. [45] integrated vision-based tactile sensing into a fin-ray soft gripper to predict fruit firmness through deformation analysis using deep learning. Wei et al. [46] developed robotic electronic skin arrays to identify occluded fruit types. Such sensor-integrated robotic platforms enable the robot to physically interact with fruits, allowing for multimodal perception beyond vision, thereby improving crop classification accuracy. Therefore, embedding stretchable sensors into robotic systems and combining them with machine learning algorithms holds strong promise for real-time crop species identification and smart harvesting applications.

**Fig. 1.**
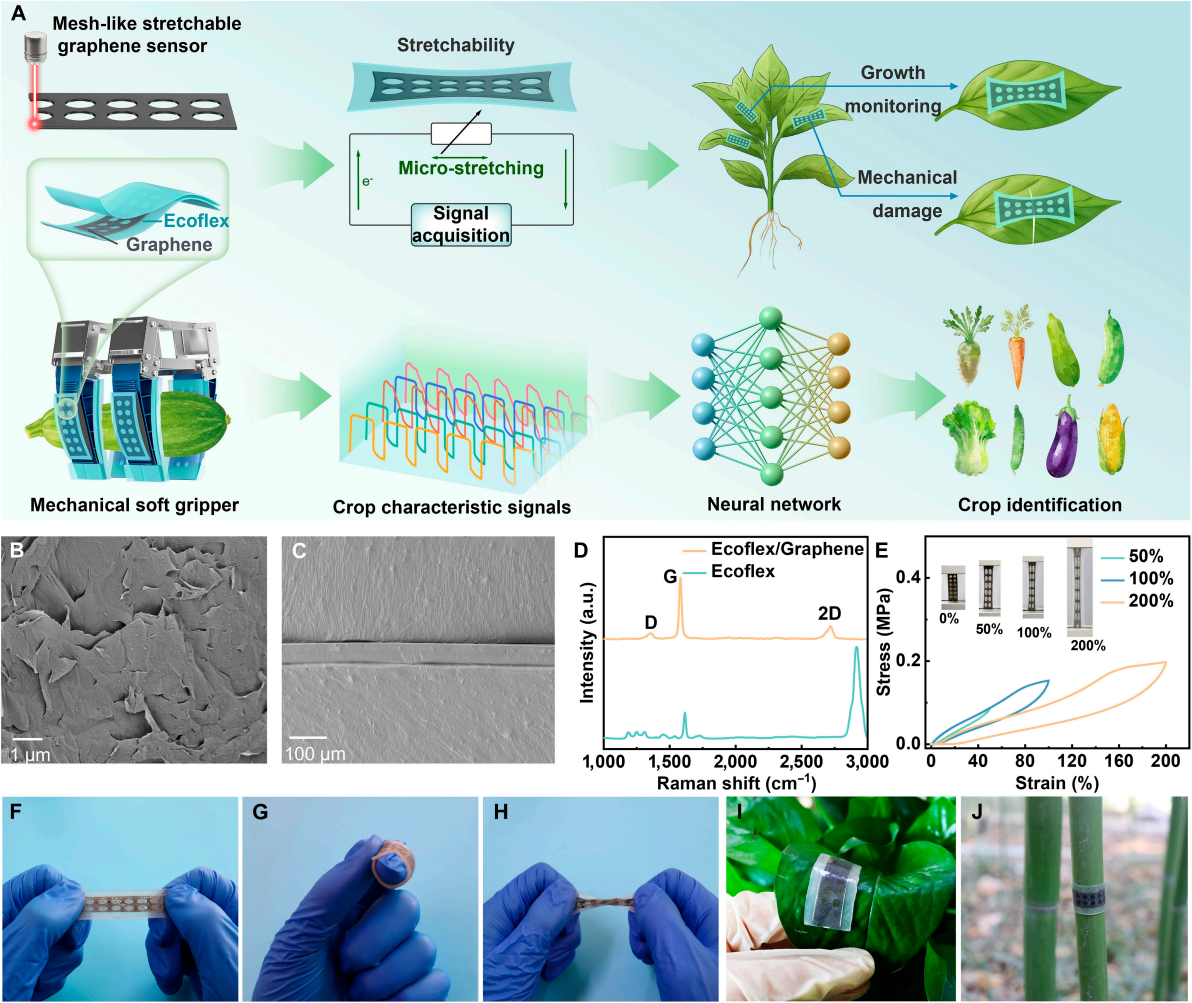
(A) Overview of the mesh-like graphene strain sensor. (B) SEM image of the graphene surface. (C) Cross-section SEM image of the stretchable sensors. (D) Raman spectra of Ecoflex and Ecoflex–graphene composite materials. (E) Stress–strain curves of the mesh-like stretchable sensor under 50%, 100%, and 200% tensile strain. (F to H) Schematic diagram showing the ability of mesh-like graphene sensors to undergo stretching, curling, and twisting. Schematic diagram of the mesh-like stretchable sensor applied to (I) the leaf and (J) the stem of plants.

In this work, we designed and developed a highly stretchable and reliable mesh-like strain sensor for plant health monitoring and crop recognition in Fig. 1A. The strain sensor was based on a graphene/Ecoflex composite material, and the optimized mesh-like structure was successfully ablated on the surface of the graphene by using laser processing. Compared to conventional unstructured strain sensors, this mesh design substantially improves the sensing range and sensitivity, reduces the detection limit to 0.1%, and ensures accurate detection of subtle plant deformations. The effective Ecoflex encapsulation further enables the sensor to be waterproof, acid- and alkali-resistant, biocompatible, and highly stable over repeated strain cycles, which is critical for real-world agricultural environments. These characteristics allow the mesh-like graphene strain sensors to not only monitor plant growth in real time for tracking plant development patterns but also detect mechanical damage in situ for predicting various plant stresses. In addition, the sensor was integrated into a robotic arm for grasping different crops, and combined with machine learning algorithms, it achieved accurate identification of 8 crop species. This multifunctional platform not only surpasses many existing plant-wearable sensors in terms of environmental stability and operational reliability but also demonstrates superior classification performance, highlighting its potential for both wearable plant electronics and robotic-assisted precision agriculture. By combining high mechanical performance, robust environmental adaptability, and machine learning-enabled crop recognition, this work presents a unique and versatile solution for intelligent agriculture.

## Results and Discussion

### Fabrication and characteristics of the graphene-based stretchable strain sensor

The preparation process for the mesh-like stretchable strain sensors is shown schematically in Fig. S1. Ecoflex served as the substrate and encapsulation layer, whereas the graphene film acted as the sensing layer. The graphene film was designed with a mesh-like structure to enhance the mechanical stability of sensors. Figure 1B exhibits the surface morphology of graphene composite materials, in which an irregular layered structure on the surface can be clearly observed. A cross-sectional scanning electron microscopy (SEM) image of the mesh-like graphene sensor is presented in Fig. 1C, indicating strong bonding between the graphene material and the Ecoflex elastic substrate. Raman spectroscopy was applied to determine the Ecoflex–graphene composite quality. As shown in Fig. 1D, compared to the featureless Raman signal of the Ecoflex film, the graphene film exhibits 2 similar peaks representing the graphene intrinsic features. The D peak is located near 1,350 cm^−1^ and is caused by defects and structural disturbances in the graphene. The G peak observed near 1,580 cm^−1^ is due to vibrations of sp2 hybridized carbon atoms in the 2-dimensional hexagonal lattice [47,48]. The stress–strain curve of the Ecoflex–graphene composite is shown in Fig. S2. The composite exhibited a large elongation of approximately 700% and its tensile strength reached 0.6 MPa. Figure 1E shows the entire continuous stretching and releasing process of the sensor from 50% to 200% strain, showing that the 50% and 100% strain–release curves exhibited good recovery properties, while the 200% strain–release curve showed marked hysteresis, which was attributed to the creep behavior of Ecoflex [38]. Owing to the super-elasticity of Ecoflex, the mesh-like graphene sensor displayed an excellent mechanical performance and was capable of being stretched, rolled, and twisted (Fig. 1F to H). Figure S3 shows the transparency of the mesh-like graphene sensor, through which the letters “HAU” are visible. In addition to its super-elasticity, Ecoflex also exhibits good biocompatibility, allowing the sensor to be directly attached to the plant surface for plant growth monitoring without the need for any additional adhesives (Fig. S4). As shown in Fig. 1I to J, when this sensor was attached to the *Epipremnum aureum* leaf surface and bamboo stem, respectively, they demonstrated good conformity with the plant leaf and stem.

### Structure optimization of graphene-based stretchable strain sensor

**Fig. 2.**
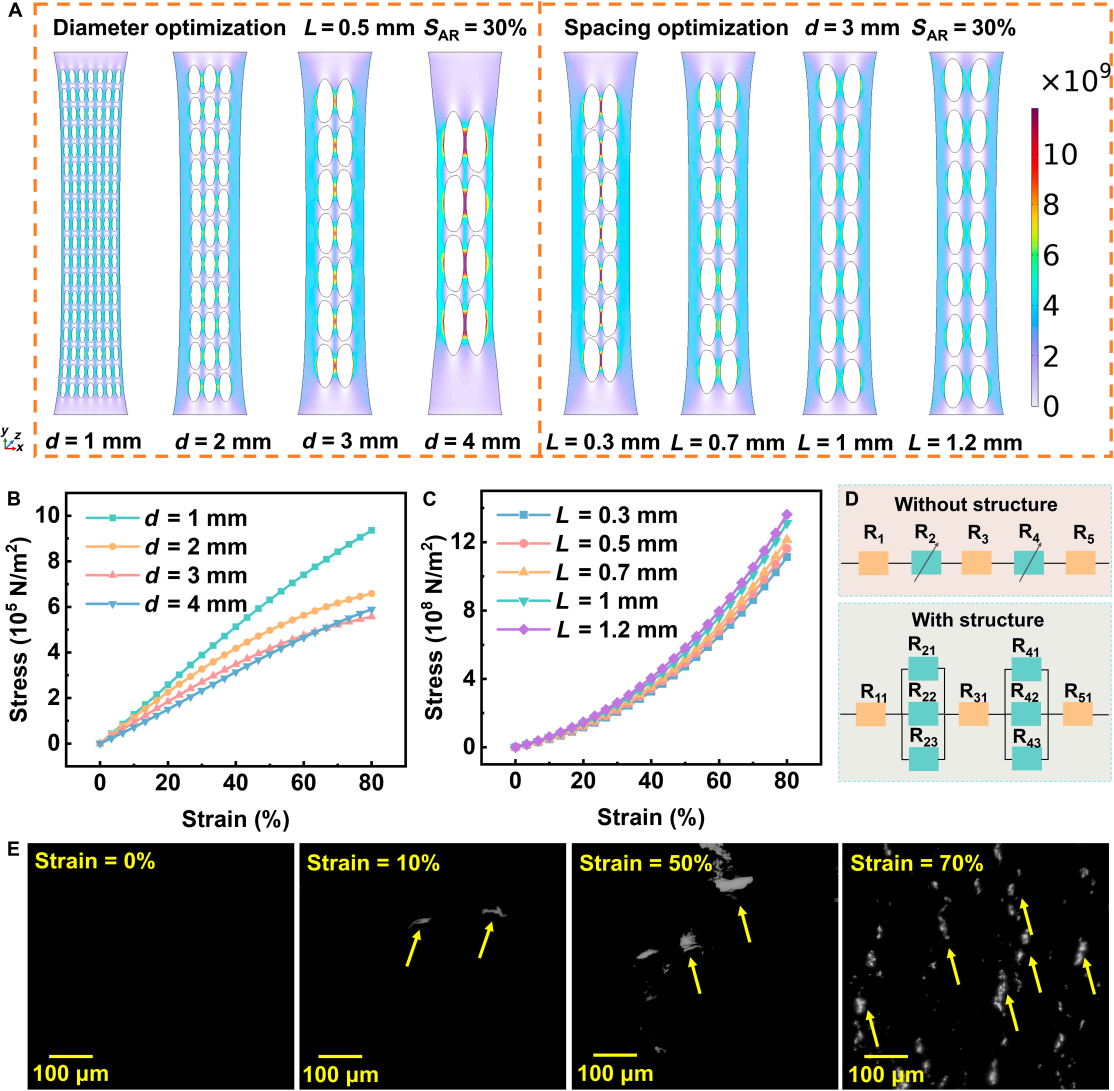
Optimization simulation of the mesh-like stretchable sensor. (A) Simulation results of the stress–strain distribution. (B) Optimization of the diameter of the circles. (C) Optimization of the spacing between the circles. (D) Equivalent circuit diagram of the mesh-like structure. (E) Optical microscopy images of mesh-like graphene sensors at a strain of 0%, 10%, 50%, and 70%, respectively.

The dimensions of the mesh-like structure were optimized by using finite element simulation analysis. Figure S5 shows 8 different mesh-like models used for comparison. The total sensing area was fixed at 330 mm^2^, and the circular openings in each design occupied about 30% of that area (*S*_AR_) (Tables S1 and S2). This approach allowed us to adjust the diameter (*d*) and spacing (*L*) of the circle while keeping the same overall occupied area, thereby eliminating the interference effects caused by different numbers of holes. Figure 2A shows the simulation results of the stress–strain distribution. Under the same tensile strain, stress was concentrated around the mesh structure. The stress concentration is more pronounced around mesh structures with larger circular diameters and smaller circular spacings. Therefore, by reducing the local stress concentration, the strain range that can be tolerated was increased, thereby enhancing the sensor’s durability and stability under large tensile strains. The strain (*Ɛ*) is defined as *Ɛ* = *L*_s_/*L*_0_ × 100%, where *L*_s_ refers to the length of the sensor displacement during the stretching process and *L*_0_ represents the initial length of the sensor. The stress (*σ*) is calculated as *σ* = *F*/*A*_0_*,* where *F* is the tensile force applied to the sensor during stretching and *A*_0_ is the effective sensing area of the entire sensor. As shown in Fig. 2B, when the strain of the sensor reached 80%, the tensile stress gradually decreased as the diameter of the circles increased. This indicates that increasing the size of the circular structure helps disperse the stress, thereby reducing the tensile stress on the material. Figure 2C shows that as the spacing increases, the tensile stress decreases slightly. However, when the spacing was too small, stress during the stretching process concentrated at the connection points between adjacent circles, which heightened the risk of graphene fracture and diminished the overall sensing performance of the mesh-like graphene sensor.

Figure 2D illustrates the equivalent circuit of the stretchable sensor. In the absence of a mesh structure, the resistance of the entire graphene conductive layer was equivalent to that of a series resistance, resulting in a higher resistance. Additionally, during the stretching process, the change in resistance was minimal, leading to low sensor sensitivity. If any conductive pathway breaks, the entire sensor enters an open-circuit state. In contrast, when the sensor incorporated a mesh structure, the resistance of the graphene conductive layer consisted of a parallel combination of the resistances of the structured and nonstructured areas. In this case, the overall resistance of the sensor was relatively low and the mesh structure enhanced the conductive pathways within the conductive layer throughout the stretching process. As shown in Fig. 2E, at 0% strain, the graphene microstructure remains dense and continuous. At 10% strain, small cracks start to form in the graphene surface, but these cracks do not materially alter the conductivity of the material. At 50% strain, the number and size of cracks increase, causing a more substantial disruption of the conductive pathways, which will increase the resistance of the sensor. At 70% strain, the cracks dramatically expand, and large-scale rupture of the conductive pathways occurs. The material experiences a failure in tunneling conduction, and the gaps between the graphene sheets increase, creating additional resistance to electron flow [49]. Figure S6 presents SEM images of the graphene surface after removing the encapsulating Ecoflex material at 0%, 10%, 50%, and 70% strain. Although crack density and size appear relatively higher without encapsulation due to the absence of Ecoflex constraint, the overall trend in crack initiation, propagation, and enlargement with increasing strain is consistent with the observations in the encapsulated sensor (Fig. 2E).

### Performance of graphene-based stretchable strain sensor

**Fig. 3.**
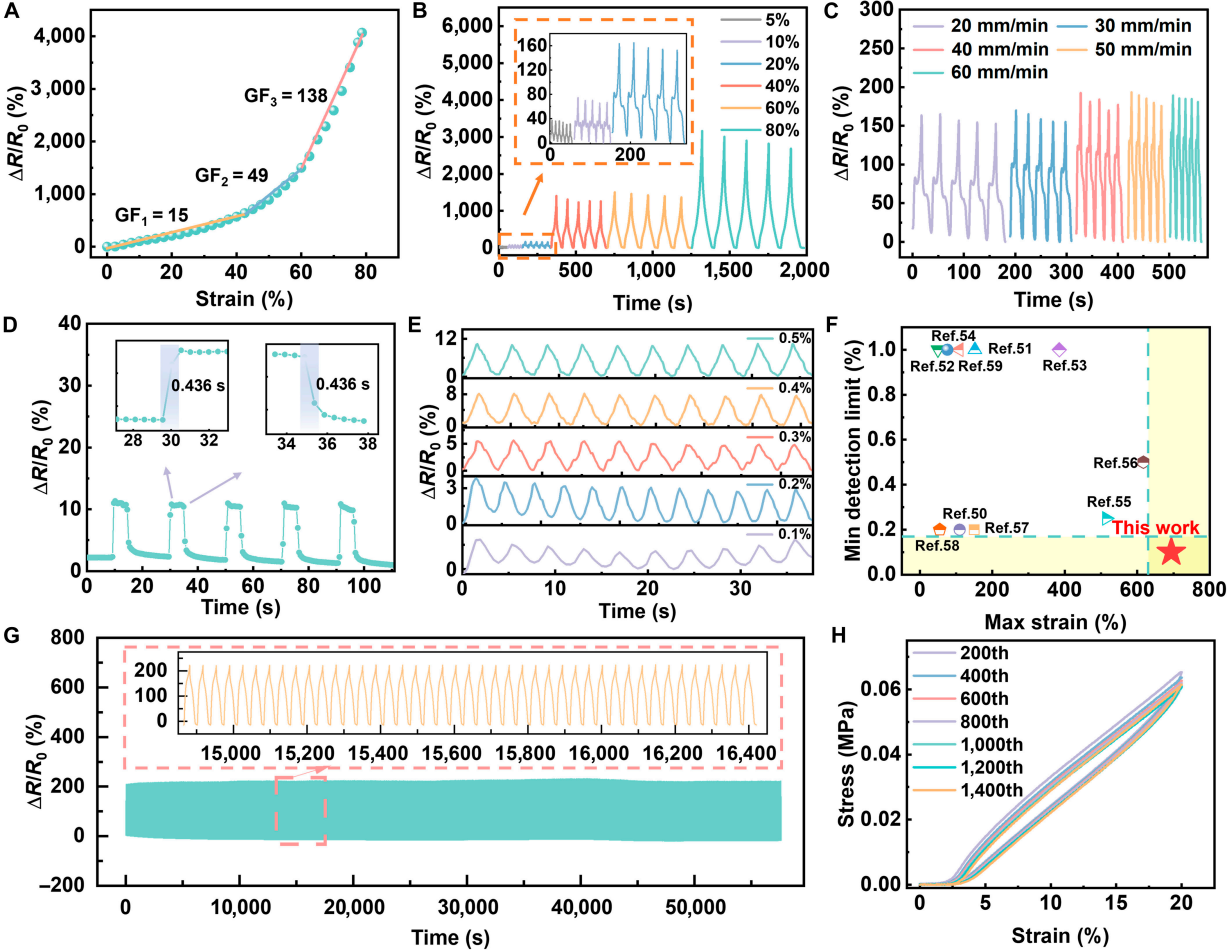
Performance of the mesh-like stretchable sensors. (A) Gauge factor (GF) curve of the mesh-like stretchable sensor under different strains. (B) Relative resistance change rate of the mesh-like stretchable sensor under different strains. (C) Relative resistance change rate of the mesh-like stretchable sensor during a 20% tensile strain at different loading speeds. (D) Dynamic response curve of the mesh-like stretchable sensor showing response time and recovery time. (E) Minimum detection limit of 0.1% for the mesh-like stretchable sensor. (F) A comparison of the minimum detection limit between the work with the reported wearable sensors. (G) Resistance change rate curve of the mesh-like graphene sensor after over 1,500 tensile release cycles under 20% strain. (H) Stress–strain curves at the 200th, 400th, 600th, 800th, 1,000th, 1,200th, and 1,400th cycles.

The strain GF of the mesh-like stretchable sensor is defined as GF = (Δ*R*/*R*_0_)/*ε*, where *ε* is the strain change, Δ*R* is the resistance change, and *R*_0_ is the initial resistance. As shown in Fig. 3A, the GF of the mesh-like stretchable sensor can be divided into 3 linear regions, each corresponding to a distinct GF value. The first region, ranging from 0% to 40% strain, has a GF value of 15. The second region, from 40% to 60% strain, corresponded to a GF value of 49, and the third region, from 60% to 80% strain, exhibited a GF value of 138. At small strains, the morphological changes in the sensor were minimal, leading to a small variation in the resistance. With the strain increased, the stress concentrated around the mesh structure, causing it to deform and enhancing the relative resistance change rate of the sensor. When the strain reached a certain level, cracks formed between the mesh structures owing to the concentrated stress, substantially increasing the resistance change and thereby improving the sensor sensitivity. In addition to uniaxial stretching, the performance of the sensor under bending and torsion was also evaluated. As shown in Fig. S7, the relative resistance change rate remained within 2% when the bending angle ranged from 0° to 50°, and as shown in Fig. S8, within the same range, the relative resistance change rate of the torsion angle remained within 0.5%. These confirm that the sensor maintains excellent electrical stability even under complex deformation modes commonly found in natural plant environments, ensuring reliable signal acquisition in practical applications. Under different cyclic tensile strains, the sensor exhibited a relatively stable resistance change rate, indicating excellent dynamic stability (Fig. 3B). At a strain of 20%, the variation in resistance of the mesh-like graphene stretchable sensor remained relatively stable at different strain rates (Fig. 3C). Figure 3D shows the response and recovery times of sensors under rapid stretching, indicating that the mesh-like stretchable sensor can respond quickly to external stimuli. The mesh-like stretchable sensor had a minimum detection limit of 0.1% strain (Fig. 3E). Due to the slow growth of plants and their relatively minor changes, the mesh-like stretchable sensors can be used to monitor plant growth. Figure S9 shows a strong synchronization between the resistance change and the applied tensile strain. Figure 3F compares the mechanical performance of the mesh-like stretchable sensors with that of wearable sensors reported in the literature, demonstrating the outstanding overall mechanical properties of the sensor developed in this work [50–59]. Cyclic tests were performed to evaluate the reliability of mesh-like stretchable sensors. As can be seen from Fig. 3G, the mesh-like stretchable sensor subjected to a 20% strain still exhibited a stable tensile response after more than 1,500 cycles of testing. This indicates high repeatability and supports the use of wearable sensors for plant health monitoring. As illustrated in Fig. 3H, the sensor exhibited almost no marked hysteresis during each stretching–release cycle, indicating good synchronization between the resistance change and tensile strain. Figure S10 illustrates the change in the resistance of mesh-like stretchable sensors over 8 days. The curve showed that the resistance of the sensors remained relatively stable without considerable changes, further confirming the long-term stability of the mesh-like stretchable sensor.

### Graphene-based stretchable strain sensors for in situ plant growth monitoring

**Fig. 4.**
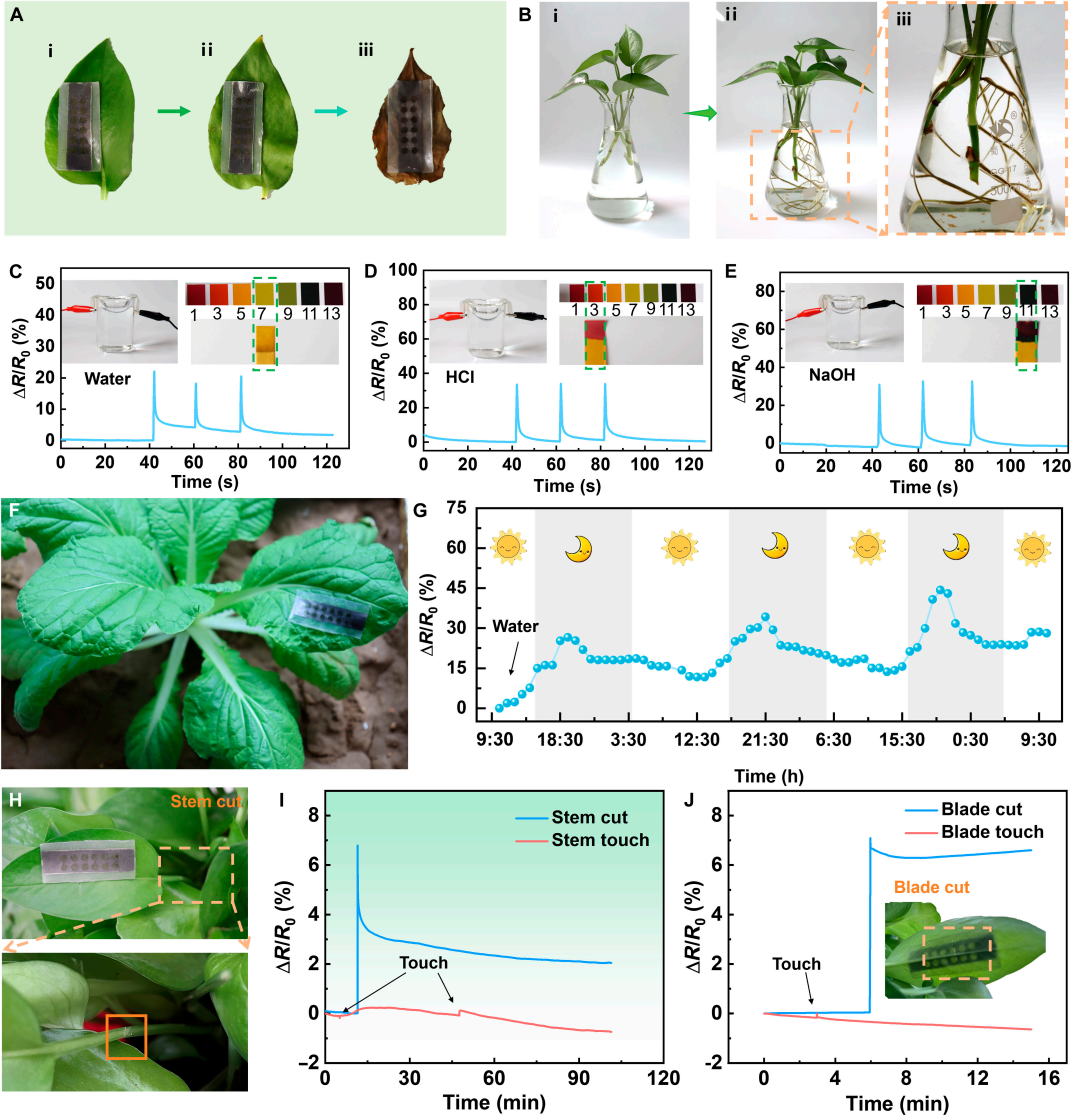
Biocompatibility and plant-grown monitoring of the mesh-like stretchable sensor. (A) Sequential changes in the mesh-like stretchable sensor adhered to *Epipremnum aureum* leaf over 1 month. (B) Growth of the *E. aureum* leaf 40 days after the mesh-like stretchable sensor adhered to the leaf. The relative resistance change rate of the mesh-like stretchable sensor in (C) water, (D) acidic, and (E) alkaline environments. (F) Schematic diagram of the mesh-like stretchable sensor adhered to the surface of a *Brassica chinensis* leaf. (G) The relative resistance change rate of the mesh-like stretchable sensor for monitoring the *B. chinensis* growth over 3 days. (H) Photos of mechanical damage to the stem of the *E. aureum*. (I) The relative resistance change rate of the sensor in response to mechanical damage at the stem of the *E. aureum*. (J) Photo of mechanical damage to the *E. aureum* leaf and the corresponding relative resistance change rate of the sensor.

Biocompatibility is a prerequisite for wearable sensors in plant applications, particularly those in direct contact with plants. As shown in Fig. 4A, the mesh-like sensor was attached to the surface of a freshly harvested *E. aureum* leaf, and observations were made over 1 month. Figure 4A(i) exhibits the sensor affixed to the surface of a fresh leaf on the first day, whereas Fig. 4A(ii) presents the sensor applied to the leaf surface 1 week later. Figure 4A(iii) shows the sensor on the leaf surface after 1 month. The results indicated that, regardless of whether the leaf was fresh or wilted, the mesh-like stretchable sensors adhered well to the leaf surface. To further confirm the biocompatibility of the mesh-like stretchable sensor, several cuttings of *E. aureum* were grown hydroponically and the sensor was attached to one of the leaves (Fig. 4A,i). After 40 days, Fig. 4B(ii) clearly shows the development of a large root system in the plant (Fig. 4B,iii is a close-up view). Compared to the leaf condition 40 days prior, the leaves did not show any signs of yellowing and were larger and healthier. These observations suggest that the mesh-like stretchable sensors do not interfere with the normal growth of the plant, nor do they affect photosynthesis, which is crucial for plant growth, as it provides the energy and nutrients required for development. Therefore, the mesh-like stretchable sensor demonstrates excellent biocompatibility.

The nature of climatic conditions poses another inevitable challenge for wearable sensors in plant applications. As exhibited in Fig. 4C to E, the sensor was placed in water, hydrochloric acid (HCl), and sodium hydroxide (NaOH) solutions. Every 20 s, the surface of the sensor was gently touched with tweezers, and the process was repeated 3 times. The relative resistance change rate of the sensor remained stable throughout the experiment. When the sensor was continuously soaked in an alkaline solution for 1 h without any mechanical deformation (Fig. S11), the sensor exhibited a resistance change rate of less than 2% throughout the entire immersion period, demonstrating its excellent chemical stability in alkaline environments. This indicates that the mesh-like graphene strain sensor encapsulated in Ecoflex possesses excellent waterproofing and chemical resistance. This is due to the Ecoflex layer not only protecting the sensor from water and corrosive environments but also ensuring that it forms a compliant and stable adhesion with the plant surface, thereby enabling reliable strain detection in complex outdoor environments [60].

In addition to chemical stability, the environmental adaptability of the strain sensor was further evaluated under various ambient conditions, including temperature, humidity, ultraviolet (UV) exposure, illumination, and wind speed. As shown in Figs. S12 and S13, the strain sensor maintains stable electrical response under moderate environmental fluctuations, including temperatures ranging from 10 to 50 °C, relative humidity levels from 35% to 75%, UV intensity from 40 to 350 mW cm^−2^ (365 nm), illumination from 0 to 250 μmol m^−2^ s^−1^, and wind speeds up to 6 m/s. These demonstrate that the sensor maintains reliable performance in typical outdoor agricultural environments, proving its suitability for long-term plant monitoring applications.

Figure 4F shows that the sensor was firmly attached to the *Brassica chinensis* leaf surface for a continuous 3-day in situ growth monitoring experiment. The plants were watered thoroughly before starting the measurement to ensure consistent growth conditions. Figure 4G and Fig. S14 present the diurnal strain variation patterns obtained during this period and the corresponding environmental temperature and humidity. It is observed that the growth patterns are consistent across the 3 days, with growth occurring predominantly at night while daytime growth was relatively slow. This is because plants absorb light during the day for photosynthesis and store energy, which is then utilized for growth at night [61]. Moreover, to evaluate its practical applicability, the sensor was deployed in outdoor agricultural fields to monitor the growth of eggplant fruits over 2 h (Fig. S15). It is evident that the growth of the eggplant is not continuous but follows a stepwise pattern. This growth behavior is similar to that observed in *Cucurbita pepo* [62]. To test plant stress, a scalpel was used to induce mechanical damage to the leaf and stem of the *E. aureum* (Fig. 4H to J). Immediately after cutting, the resistance values changed substantially, then gradually stabilized over time. In contrast, slight manual touching of the leaves or stems caused only negligible changes in sensor resistance. It indicates that mesh-like stretchable sensors can sensitively and specifically detect mechanical damage in plant tissues, in addition to their capability for monitoring plant growth.

### Deep learning-assisted stretchable strain sensor for crop recognition

**Fig. 5.**
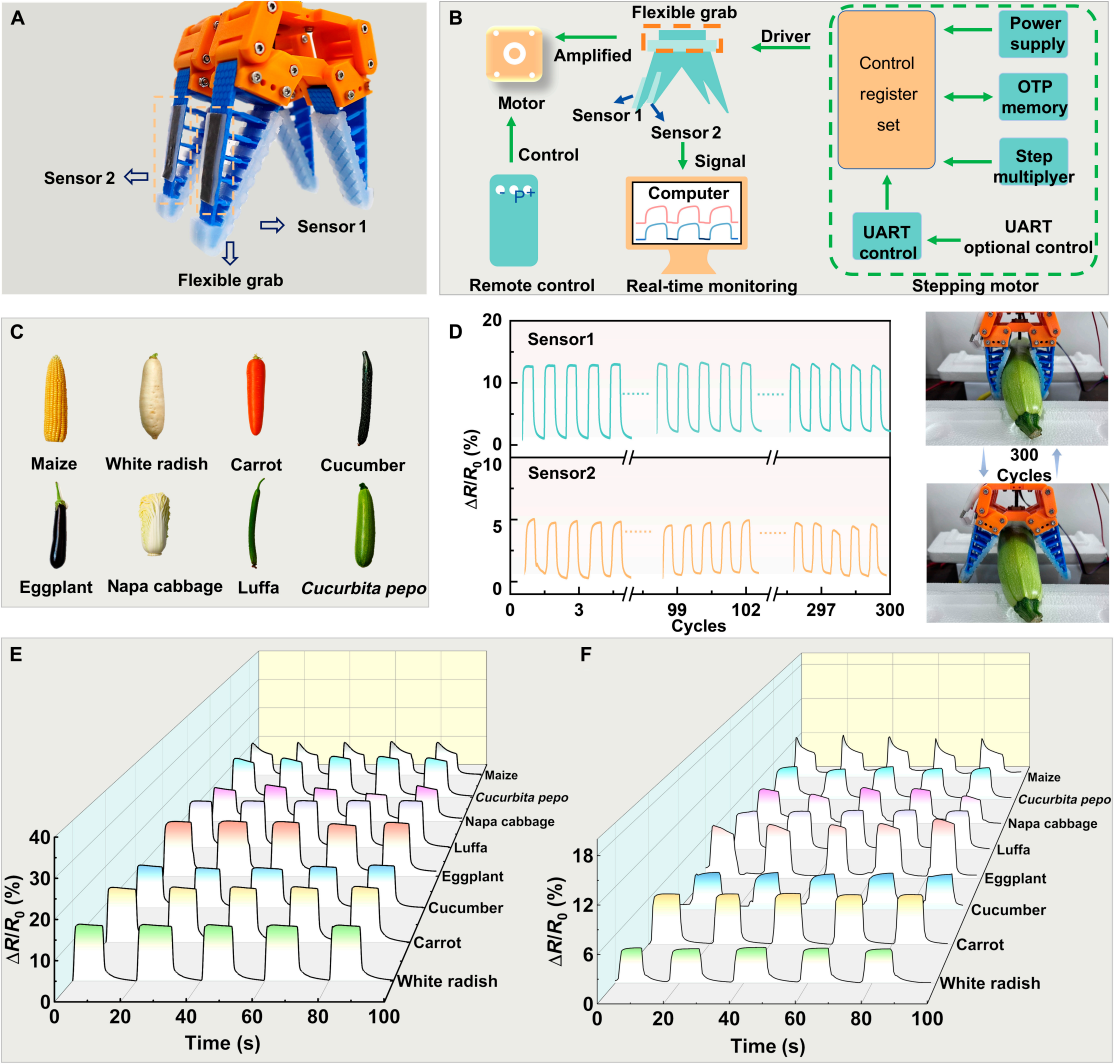
Machine learning signal acquisition. (A) Schematic diagram of the flexible gripper and sensor attachment position. (B) Driving steps of the flexible gripper. (C) Illustrations of the 8 types of crops. (D) Photos of the gripping and releasing cycle. (E and F) Partial response curves of sensor 1 and sensor 2 for the 8 crops.

A flexible gripper can deform in a coordinated manner to conform to the shape of the object it grasps, thereby making it functionally similar to a simple soft robot. Two mesh-like stretchable sensors were integrated onto the outer surface of a 4-finger flexible gripper, enabling it to perform sensing capabilities through close contact. As shown in Fig. 5A, the 2 mesh-like stretchable sensors were placed on the same side of the 2 gripper fingers and were labeled as sensor 1 and sensor 2. The flexible gripper was driven by a stepper motor for opening and closing, and data from sensor 1 and sensor 2 were collected via a dual-channel system. The control process is illustrated in Fig. 5B. Figure 5C shows 8 different crops: Maize (*Zea mays*), white radish (*Raphanus sativus*), carrot (*Daucus carota*), cucumber (*Cucumis sativus*), eggplant (*Solanum melongena*), luffa (*Luffa acutangular*), napa cabbage (*Brassica rapa*), and *C. pepo*. The signal responses from different parts of the crop were obtained using the front and rear sensors, and the process was repeated 300 times to collect a large dataset of sensor signals for machine learning. Figure 5D displays the signal response from gripping and releasing *C. pepo*, along with the pictorial representation. Figures S16 and S17 present the signal responses over 300 cycles for 7 other crops, as well as illustrations of the gripping and releasing actions of the flexible gripper. These response signals further demonstrate the excellent stability of the sensor. The data for the 8 crops are partially shown in Fig. 5E and F, where Fig. 5E represents the signal response from sensor 1, and Fig. 5F represents the signal response from sensor 2. Because of the different positions of the 2 sensors, the obtained signal responses varied. However, the response signals for the same crop remained relatively stable across each cycle. This stability is beneficial for data analysis and facilitates the extraction of feature values for different crops, which is essential for subsequent model training.

**Fig. 6.**
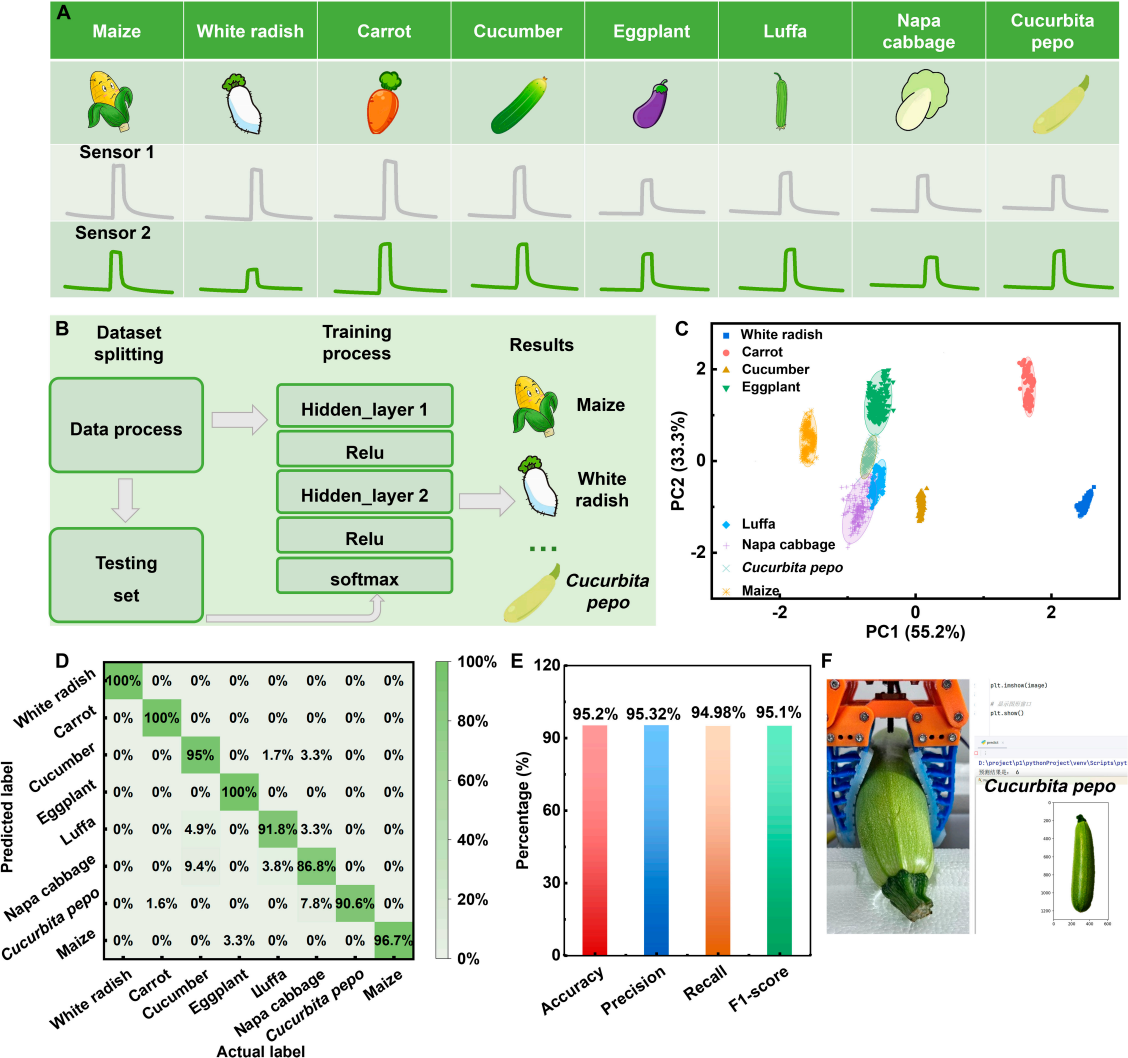
Machine learning results and one-period prediction. (A) Prediction data for 8 types of crops and their one-period forecast. (B) Machine learning process for crop identification using the mesh-like stretchable sensors. (C) Two-dimensional clustering diagram of the crop identification process. (D) Confusion matrix results for crop identification. (E) Accuracy, precision, recall, and F1-score for identifying different crops by the MLP model. (F) Schematic diagram of the one-period prediction process and the corresponding code.

To further assess the accuracy of the prediction results, additional data were collected for one complete cycle. Figure 6A shows the signal curves for the 8 crops over a single cycle. The data collected from the dual channel were then divided into training and test sets, where 80% was used for training and 20% was used for testing. Raw data were input into a classifier for training, and crop predictions were made using the test data (Fig. 6B). Figure 6C and D display the 2-dimensional classification results of the feature data and the corresponding confusion matrix. The results showed a clear distinction between the various crops. To comprehensively evaluate the classification performance of the proposed deep learning model for crop identification, several metrics were calculated, including accuracy, precision, recall, and F1-score [63]. As shown in Fig. 6E, the model achieved an overall accuracy of 95.2%, with a precision, recall, and F1-score of 95.32%, 94.98%, and 95.10%, respectively, across all 8 crop categories. These results indicate the robustness and balanced performance of the classifier in distinguishing between different crop types. The left panel of Fig. 6F illustrates the data collection process for one cycle. The data for one cycle of *C. pepo* were input and displayed an image of *C. pepo* in real time, confirming the accuracy of the model for crop identification. Figures S18 and S19 present the predicted response signal curves for one cycle of the 8 crops. The ability of the model to accurately identify the 8 types of crops is demonstrated in Movie S1, which shows the exceptional recognition capability of the mesh-like stretchable sensors.

## Conclusion

In summary, a highly stretchable and reliable graphene-based strain sensor with optimized mesh-like structures was developed for real-time plant health monitoring and crop identification. The strain sensors are made of graphene/Ecoflex composite materials, which have been successfully ablated to create a mesh-like structure on the graphene surface using laser processing technology. This design effectively improved the stretchability and stability of the sensor. The graphene-based strain sensor exhibits a high strain GF of 138, a low detection limit of 0.1%, and excellent reliability. Furthermore, by encapsulating the mesh structure with Ecoflex silicone, the stretchable strain sensor adheres to the surface of plant leaves without harming them or affecting normal plant growth, demonstrating excellent biocompatibility. Meanwhile, when the stretchable strain sensor was exposed to water, acidic, or alkaline solutions, there was no substantial degradation in sensor performance, which demonstrates that the sensor still functions properly in extreme natural environments. This sensor uses a unified sensing principle, strain-induced resistance variation, together with the structural benefits of its mesh architecture, giving it high sensitivity, large stretchability, and excellent environmental stability. The combination of this structural design and sensing mechanism also ensures versatility, enabling reliable strain detection in a variety of plant tissues and under a variety of deformation modes. Therefore, this sensor was well-suited for plant-wearable devices, enabling not only real-time monitoring of plant growth for capturing the pattern of diurnal variation, but also in situ detection of mechanical damage for effective prediction of plant stress. Furthermore, by integrating the strain sensor with a 4-finger flexible gripping clip and combining it with machine learning algorithms, the strain sensor accurately recognized 8 different crop types with 95.2% accuracy. The high accuracy in the crop identification and prediction cycle for different crop types was also further demonstrated. This work provides an effective solution for monitoring plant health, which will contribute to the rapid development of plant wearable electronic devices. In the future, we will focus on improving sensor integration with wireless communication, extending monitoring periods, and enhancing model adaptability to varied crops and environments. These advancements will contribute to the development of smart agriculture and autonomous crop management technologies.

## Materials and Methods

### Preparation of Ecoflex elastomers

The stainless steel was cut into 90 mm × 25 mm pieces using a laser marking machine (FP-GX30WD, Jinan Feipu), and the surface was treated with alcohol. Ecoflex components A and B (Smooth-On, USA) were blended in a 1:1 ratio by weight, agitated, and degassed to obtain the Ecoflex liquid. The stainless-steel sheet was put into a spin coater (KW-4A, Beijing SetCas), where a suitable amount of Ecoflex liquid was applied and spin-coated at a speed of 70,000 rpm for 6 s. The Ecoflex liquid was then cured at room temperature for 4 h to obtain the Ecoflex elastomer.

### Preparation of Ecoflex–graphene composites

The stainless steel was cut into thin pieces of 100 mm × 30 mm using a laser marking machine with a 40 mm × 10 mm rectangular mask cut at the center. The graphene dispersion was evenly sprayed onto a rectangular area using a spray gun (0.2 mm diameter, KOMAX Zhejiang), and after natural drying, the Ecoflex–graphene composite was obtained.

### Preparation and encapsulation of mesh-like graphene-based stretchable sensor

A mesh-like structure composed of a 2 × 6 array of circles was designed using laser marking software. A laser marking machine was then used to ablate the Ecoflex–graphene composite material according to the designed pattern, resulting in a mesh-like graphene film. The laser parameters were set to a speed of 2,000 mm/s, a power of 6 W, and a frequency of 25 kHz. Silver paste was mixed with acetone in a mass ratio of 3:5 and magnetically stirred for 5 to 10 min at a stirring rate of 3,000 rpm to obtain Ag electrode material. The Ag electrode material was then loaded into a 0.2-mm-diameter airbrush gun and evenly sprayed onto a rectangular area in the middle of a stainless-steel mask plate to obtain Ag electrodes of stretchable sensors. The Ecoflex elastomer then tightly adhered to the surface of the mesh-like graphene film and vacuum pumping was applied to encapsulate the graphene-based stretchable sensor.

### Characterizations

The surface and cross-sectional morphologies of the Ecoflex–graphene composite materials were characterized by means of SEM (ZEISS Sigma 300, Germany). A Raman spectrometer (HORIBA LabRAM HR Evolution, Japan) was used to characterize the Raman spectra of the Ecoflex–graphene composite material and Ecoflex films. The tensile mechanical properties of the sensor and its performance under bending and twisting were characterized using a micro-controlled universal testing machine (UTM2103, Shenzhen Sansi Zongheng Technology Co., Ltd., China). Optical images were obtained using a metallographic microscope (NJF120A). Illumination, temperature, and humidity stability were tested by an artificial climate chamber (HP100GS-LED). During the tensile test, the signal from the mesh-like graphene sensor was measured using a digital multimeter (DMM6500; Keithley, USA).

### Finite element analysis simulation

To determine the optimal size of the mesh-like structure, COMSOL Multiphysics software was used to create a 3D model of the mesh-like graphene sensor for finite element analysis. The following basic parameters were used to simulate the sensor model: Ecoflex substrate with a Poisson’s ratio of 0.49, a Young’s modulus of 2 MPa, and a density of 970 kg/m^3^; graphene with a Poisson’s ratio of 0.16, a Young’s modulus of 1 × 10^9^ Pa, a conductivity of 1 × 10^8^ S/m, and a density of 790 kg/m^3^.

### Deep learning-based data analytics

In this work, a multilayer perceptron (MLP) model was developed using Python to classify crop species. The network consisted of an input layer, 2 hidden layers with 5 and 8 neurons, respectively (both activated by ReLU), and a Softmax output layer. Before the algorithm training, a 4-finger flexible gripper was used with sensors placed on 2 grippers on the same side. Eight crop species were selected for the experiment: maize, white radish, carrot, cucumber, eggplant, luffa, napa cabbage, and *C. pepo*. For each crop, 300 grasping cycles were performed, each lasting 20 s, resulting in a total of 2,400 labeled samples. The raw time-series data were normalized and converted into fixed-length feature vectors. The dataset was divided into training (80%) and testing (20%) sets, ensuring no overlap of plant individuals between sets. Model training was performed using the Adam optimizer with a learning rate of 0.001, a batch size of 200, and a maximum of 200 epochs. The loss function was categorical cross-entropy, and early stopping was applied to avoid overfitting. After training, an additional independent cycle of data was used for model inference and evaluation.

## Data Availability

All data are available in the main text or the Supplementary Materials. Source data are available from the corresponding authors upon reasonable request.

## References

[1] Zhong D, Wu C, Jiang Y, Yuan Y, Kim M-G, Nishio Y, Shih C-C, Wang W, Lai J-C, Ji X, et al. High-speed and large-scale intrinsically stretchable integrated circuits. Nature. 2024;627(8003):313–320. 38480964 10.1038/s41586-024-07096-7

[2] Kim D, Zarei M, Lee S, Lee H, Lee G, Lee SG. Wearable standalone sensing systems for smart agriculture. Adv Sci. 2025;12(16):2414748. 10.1002/advs.202414748 PMC12021045 40125565

[3] Zhou S, Zhou J, Pan Y, Wu Q, Ping J. Wearable electrochemical sensors for plant small-molecule detection. Trends Plant Sci. 2024;29(2):219–231. 38071111 10.1016/j.tplants.2023.11.013

[4] Chen H, Zhou S, Chen J, Zhou J, Fan K, Pan Y, Ping J. An integrated plant glucose monitoring system based on microneedle-enabled electrochemical sensor. Biosens Bioelectron. 2024;248: Article 115964. 38160635 10.1016/j.bios.2023.115964

[5] Li W, Kong L, Xu M, Gao J, Luo L, Li Y, Wang K, Zhou Y, Li L, Zhang X, et al. Microsecond-scale transient thermal sensing enabled by flexible Mo_1−x_W_x_S_2_ alloys. Research. 2024;7:0452. 39171118 10.34133/research.0452 PMC11337116

[6] Zhou J, Fan P, Zhou S, Pan Y, Ping J. Machine learning-assisted implantable plant electrophysiology microneedle sensor for plant stress monitoring. Biosens Bioelectron. 2025;271: Article 117062. 39708493 10.1016/j.bios.2024.117062

[7] Kim D, Won J, Park H, Choi JG, Park S, Lee G. Toward the 3rd generation of smart farming: Materials, devices, and systems for e-plant technologies. Adv Funct Mater. 2025; Article e12264.

[8] Li X, Li M, Li J, Gao Y, Liu C, Hao G. Wearable sensor supports in-situ and continuous monitoring of plant health in precision agriculture era. Plant Biotech J. 2024;22(6):1516–1535. 10.1111/pbi.14283 PMC11123445 38184781

[9] Yin J, Jia P, Ren Z, Zhang Q, Lu W, Yao Q, Deng M, Zhou X, Gao Y, Liu N. Recent advances in self-powered sensors based on ionic hydrogels. Research. 2025;8:0571. 39810855 10.34133/research.0571 PMC11729273

[10] Zhang Q, Ying Y, Ping J. Recent advances in plant nanoscience. Adv Sci. 2022;9(2):2103414. 10.1002/advs.202103414 PMC8805591 34761568

[11] Alqaderi AIJ, Ramakrishnan N. Carbon-based flexible strain sensors: Recent advances and performance insights in human motion detection. Chem Eng J. 2025;513: Article 162609.

[12] Song C, Lee H, Park C, Lee B, Kim J, Park C, Lai C, Cho SJ. Advances in crack-based strain sensors on stretchable polymeric substrates: Crack mechanisms, geometrical factors, and functional structures. Polymers. 2025;17(7):941. 40219330 10.3390/polym17070941 PMC11991081

[13] Yan L, Liu Z, Wang J, Yu L. Integrating hard silicon for high-performance soft electronics via geometry engineering. Nano Micro Lett. 2025;17(1):218. 10.1007/s40820-025-01724-1 PMC11996752 40227525

[14] Tang B, Zhou J, Zhao C, Pan Y, Lu Y, Liu C, Ma K, Sun X, Zhang R, Gu X. Using UAV-based multispectral images and CGS-YOLO algorithm to distinguish maize seeding from weed. Artif Intell Agric. 2025;15(2):162–181.

[15] Fu H, Zhao X, Tan H, Zheng S, Zhai C, Chen L. Effective methods for mitigate the impact of light occlusion on the accuracy of online cabbage recognition in open fields. Artif Intell Agric. 2025;15(3):449–458.

[16] Bourriz M, Hajji H, Laamrani A, Elbouanani N, Abdelali HA, Bourzeix F, El-Battay A, Amazirh A, Chehbouni A. Integration of hyperspectral imaging and AI techniques for crop type mapping: Present status, trends, and challenges. Remote Sens. 2025;17(9):1574.

[17] Sweet DD, Tirado SB, Springer NM, Hirsch CN, Hirsch CD. Opportunities and challenges in phenotyping row crops using drone-based RGB imaging. Plant Phenome J. 2022;5(1): Article e20044.

[18] Zheng Y, Zhang H, Li X, Zhao Y, Li Z, Hou Y, Liu C, Wang Q. Fast-zoom and high-resolution sparse compound-eye camera based on dual-end collaborative optimization. Opt Commun. 2025;8(6): Article 240285.

[19] Jiang Q, Zhao X, Zhao TY, Li WL, Ye J, Dong X, Wang X, Liu Q, Ding H, Ye ZY, et al. A machine-learning-powered spectral-dominant multimodal soft wearable system for long-term and early-stage diagnosis of plant stresses. Sci Adv. 2025;11(26): Article eadw7279. 40577461 10.1126/sciadv.adw7279 PMC12204154

[20] Chen H, Zhou J, Long X, Zhuo F, Liu Y, Zhao Y, Xie J, Duan H, Fu Y. Fibrous mats based skin sensor with ultra-sensitivity and anti-overshooting dynamic stability enhanced with artificial intelligence. Chem Eng J. 2023;473: Article 145054.

[21] Zhou J, Guo Y, Wang Y, Ji Z, Zhang Q, Zhuo F, Luo J, Tao R, Xie J, Reboud J, et al. Flexible and wearable acoustic wave technologies. Appl Phys Rev. 2023;10(2): Article 021311.

[22] Wang S, Edupulapati B, Hagel JM, Kwok JJ, Quebedeaux JC, Khasbaatar A, Baek JM, Davies DW, Ella Elangovan K, Wheeler RM, et al. Highly stretchable, robust, and resilient wearable electronics for remote, autonomous plant growth monitoring. Device. 2024;2(4): Article 100322.

[23] Xu K, Cai Z, Luo H, Lu Y, Ding C, Yang G, Yang H. Toward integrated multifunctional laser-induced graphene-based skin-like flexible sensor systems. ACS Nano. 2024;18(39):26435–26476. 39288275 10.1021/acsnano.4c09062

[24] Ding Z, Liu P, Xu K, Tang G, Zhu C, Chen J, Liu F. Facile construction of graphene based ultra folding resistance and high-efficiency electric heating film by multi-molecules induced orientation and interface regulation. Chem Eng J. 2025;508: Article 160845.

[25] Tao L, Wang D, Tian H, Ju Z, Liu Y, Pang Y, Chen Y, Yang Y, Ren T. Self-adapted and tunable graphene strain sensors for detecting both subtle and large human motions. Nanoscale. 2017;9(24):8266–8273. 28585963 10.1039/c7nr01862b

[26] Liu H, Liu C, Luo J, Tang H, Li Y, Liu H, Wu J, Han F, Liu Z, Guo J, et al. Micromesh reinforced strain sensor with high stretchability and stability for full-range and periodic human motions monitoring. InfoMat. 2024;6(4): Article e12511.

[27] Wang B, Lee B-K, Kwak M-J, Lee D-W. Graphene/polydimethylsiloxane nanocomposite strain sensor. Rev Sci Instrum. 2013;84(10): Article 105005. 24182156 10.1063/1.4826496

[28] Li X, Zhang R, Yu W, Wang K, Wei J, Wu D, Cao A, Li Z, Cheng Y, Zheng Q, et al. Stretchable and highly sensitive graphene-on-polymer strain sensors. Sci Rep. 2012;2(1):870. 23162694 10.1038/srep00870 PMC3499758

[29] Lin Z, Wang W, Liu R, Li Q, Lee J, Hirschler C, Liu J. Cyborg organoids integrated with stretchable nanoelectronics can be functionally mapped during development. Nat Protoc. 2025;20(9):2528–2559. 40140634 10.1038/s41596-025-01147-7

[30] Yang R, Tu Z, Chen X, Wu X. Highly stretchable, robust, sensitive and wearable strain sensors based on mesh-structured conductive hydrogels. Chem Eng J. 2024;480: Article 148228.

[31] Wu Z, Ding Q, Wang H, Ye J, Luo Y, Yu J, Zhan R, Zhang H, Tao K, Liu C. A humidity-resistant, sensitive, and stretchable hydrogel-based oxygen sensor for wireless health and environmental monitoring. Adv Funct Mater. 2024;34(6):2308280.

[32] Zhao Y, Yu P, Tao Y, Zhang X, Li M, Xu W, Zhao J. Long-term stability and durability of direct-ink-writing 3D-printed sensors: Challenges, strategies and prospects. Virtual Phys Prototyp. 2025;20(1): Article e2460211.

[33] Chen H, Zhou J, Cao H, Liang D, Chen L, Yang Y, Wang L, Xie J, Duan H, Fu Y. Thermo-responsive and phase-separated hydrogels for cardiac arrhythmia diagnosis with deep learning algorithms. Biosens Bioelectron. 2025;276: Article 117262. 39965416 10.1016/j.bios.2025.117262

[34] Zhang C, Kong J, Wang Z, Tu C, Li Y, Wu D, Chen F. Origami-inspired highly stretchable and breathable 3D wearable sensors for in-situ and online monitoring of plant growth and microclimate. Biosens Bioelectron. 2024;259: Article 116379. 38749288 10.1016/j.bios.2024.116379

[35] Kim S, Kang J, Lee I, Jang J, Park CB, Lee W, Bae B-S. An intrinsically stretchable multi-biochemical sensor for sweat analysis using photo-patternable ecoflex. npj Flex Electron. 2023;7(1):33.

[36] Sun M, Lu T, Zhou Y, Chen Y, Tu W, Zhang C, Ni Z, Li X, Hu T. Structural engineering and 3D printing of highly stretchable strain sensors for smart wearable systems. Chem Eng J. 2025;518: Article 164611.

[37] Sharma S, Pradhan GB, Bhatta T, Sapkota S, Teli A, Lee Y, Lim S, Park JY. A bifunctional organic hydrogel-based standalone self-powered hybrid strain sensor band for rehabilitation monitoring and human–machine interfacing. Adv Funct Mater. 2025;35(29):2424907.

[38] Lv K, Tian G, Yan Y, Zhou H, Fan Q, Liang L, Liu N, Wang D, Song Z, Xu F, et al. Stretchable carbon nanotube/Ecoflex conductive elastomer films toward multifunctional wearable electronics. Chem Eng J. 2024;500: Article 157534.

[39] Wang S, Baek JM, Lau AP, Quebedeaux JC, Leakey ADB, Diao Y. Light-stable, ultrastretchable wearable strain sensors for versatile plant growth monitoring. ACS Sens. 2025;10(5):3390–3401. 40305745 10.1021/acssensors.4c03104

[40] Yang Y, He T, Ravindran P, Wen F, Krishnamurthy P, Wang L, Lee C. All-organic transparent plant e-skin for noninvasive phenotyping. Sci Adv. 2024;10(7): Article eadk7488. 38363835 10.1126/sciadv.adk7488 PMC10871535

[41] Wang L, Chen W, Li H, Xu X, Zhang Z, Wu L, Xu J, Huang Y, Lu B. Ultrasoft, anti-dehydrated, and highly stretchable carboxymethylcellulose-based organohydrogel strain sensors for non-invasive real-time plant growth monitoring. Carbohydr Polym. 2025;364: Article 123753. 40484590 10.1016/j.carbpol.2025.123753

[42] Lee G, Hossain O, Jamalzadegan S, Liu Y, Wang H, Saville A, Shymanovich T, Paul R, Rotenberg D, Whitfield A. Abaxial leaf surface-mounted multimodal wearable sensor for continuous plant physiology monitoring. Sci Adv. 2023;9(15): Article eade2232. 37043563 10.1126/sciadv.ade2232 PMC10096584

[43] Yang N, Huang Z, He Y, Xiao W, Yu H, Qian L, Xu Y, Tao Y, Lyu P, Lyu X, et al. Detection of color phenotype in strawberry germplasm resources based on ffeld robot and semantic segmentation. Comput Electron Agric. 2024;226: Article 109464.

[44] Su J, He K, Li Y, Tu J, Chen X. Soft materials and devices enabling sensorimotor functions in soft robots. Chem Rev. 2025;125(12):5848–5977. 40163535 10.1021/acs.chemrev.4c00906

[45] Lin J, Hu Q, Xia J, Zhao L, Du X, Li S, Chen Y, Wang X. Non-destructive fruit firmness evaluation using a soft gripper and vision-based tactile sensing. Comput Electron Agric. 2023;214: Article 108256.

[46] Wei Y, Cai L, Fang H, Chen H. Fruit recognition and classification based on tactile information of flexible hand. Sens Actuator Phys. 2024;370: Article 115224.

[47] Ji J, Zhao W, Wang Y, Li Q, Wang G. Templated laser-induced-graphene-based tactile sensors enable wearable health monitoring and texture recognition via deep neural network. ACS Nano. 2023;17(20):20153–20166. 37801407 10.1021/acsnano.3c05838

[48] Guo Y, Duan Y, Gu S, Liu X, Fan Z, Pang H, Pan L. Carbon nanocoils-assisted formation of tunable pore graphene aerogels for lightweight broadband microwave absorption, thermal insulation, and antifreeze devices. Small. 2025;21(10):2412270. 10.1002/smll.202412270 39924820

[49] Gao L, Yang J, Zhao Y, Zhao X, Zhou K, Zhai W, Zheng G, Dai K, Liu C, Shen C. Multilayer bionic tunable strain sensor with mutually non-interfering conductive networks for machine learning-assisted gesture recognition. Adv Funct Mater. 2024;35(11):2416911.

[50] Zhu W, Zhang C, Lin J, Pan S, Wang Q, Liao N, Zhang M. Flexible strain sensor based on copper/graphene composite films. ACS Appl Nano Mater. 2023;7(1):358–369.

[51] Seyedin S, Uzun S, Levitt A, Anasori B, Dion G, Gogotsi Y, Razal JM. MXene composite and coaxial fibers with high stretchability and conductivity for wearable strain sensing textiles. Adv Funct Mater. 2020;30(12):1910504.

[52] Gan L, Xu Y, Cai Y, Zhou Y. A high-sensitivity flexible stretchable fiber-optic sensor based on the waveguide loss principle. IEEE Trans Instrum Meas. 2025;74:1–7.

[53] Zhang W, Cheng H, Zhang T, Yu D, Wang W. Upgrading of cotton fabrics by ionic liquid dissolving joint with wet spinning for stretchable and weavable fiber-based strain sensors. Polymer. 2025;327: Article 128344.

[54] Gong W, Liu M, Hu B, Fan L, Ye D, Xu J. Room-temperature and recyclable preparation of cellulose nanofibers using deep eutectic solvents for multifunctional sensor applications. Int J Biol Macromol. 2025;296: Article 139739. 39798755 10.1016/j.ijbiomac.2025.139739

[55] Lin Y, Yin Q, Jia H, Ji Q, Wang J. Ultrasensitive and highly stretchable bilayer strain sensor based on bandage-assisted woven fabric with reduced graphene oxide and liquid metal. Chem Eng J. 2024;487: Article 150777.

[56] Cho S-H, Lim T, Lee H-J, Kim S-Y, Suk JW. Multifunctional wrinkled nacreous all-carbon films for high-performance stretchable strain sensors and supercapacitors. J Mater Chem A. 2024;12(39):26718–26727.

[57] Hong W, Guo X, Li X, Zhang T, Zhu X, He J, Zhang R, Yang S, Shao Y, Fang Y, et al. Fishbone and nettle fiber inspired stretchable strain sensor with high sensitivity and wide sensing range for wearable electronics. Chem Eng J. 2024;492: Article 152281.

[58] Shao HQ, Wei KD, Gong T, Jia J, Tang CY, Zha XJ, Ke K, Bao RY, Zhang K, Wang Y, et al. Elastic Janus microarray film strain sensors with heterogeneous modulus and conductivity for healthcare and braille identification. Adv Funct Mater. 2024;34(30):2316134.

[59] Meng Q, Zhao L, Geng Y, Yin P, Mao Z, Sui X, Zhao M, Benetti EM, Feng X. A one-pot approach to prepare stretchable and conductive regenerated silk fibroin/CNT films as multifunctional sensors. Nanoscale. 2023;15(21):9403–9412. 37158132 10.1039/d3nr01347b

[60] Yan B, Zhang F, Wang M, Zhang Y, Fu S. Flexible wearable sensors for crop monitoring: A review. Front Plant Sci. 2024;15:1406074. 38867881 10.3389/fpls.2024.1406074 PMC11167128

[61] Chai Y, Chen C, Luo X, Zhan S, Kim J, Luo J, Wang X, Hu Z, Ying Y, Liu X. Cohabiting plant-wearable sensor in situ monitors water transport in plant. Adv Sci. 2021;8(10):2003642. 10.1002/advs.202003642 PMC8132156 34026443

[62] Tang W, Yan T, Wang F, Yang J, Wu J, Wang J, Yue T, Li Z. Rapid fabrication of wearable carbon nanotube/graphite strain sensor for real-time monitoring of plant growth. Carbon. 2019;147:295–302.

[63] Lu H, Zhang L, Jiang J, Song J, Zhou Z, Wu W, Cheng Z, Yan T, Hu H, Zhao T, et al. Pressure induced molecular-arrangement and charge-density perturbance in doped polymer for intelligent motion and vocal recognitions. Adv Mater. 2025;37(27):2500077. 40200687 10.1002/adma.202500077 PMC12243725

