# Supplementary material for: Highly Stretchable and Reliable Graphene-Based Strain Sensor for Plant Health Monitoring and Deep Learning-Assisted Crop Recognition
Source: Research (Wash D C). 2025 Oct 7;8:0933. doi: 10.34133/research.0933 (PMC12501636; doi:10.34133/research.0933)
Supplement: Supplementary 1 — Figs. S1 to S19 Tables S1 and S2 Movie S1 [file research.0933.f1.zip › Revised Supplemental Materials.docx]

Supplemental Materials

**Highly stretchable and reliable graphene-based strain sensor for plant health monitoring and deep learning-assisted crop recognition**

Yaling Wang^1^, Pan Li^1^, Zhizhao Liu^1^, Jiakun Kang^2^, Ke Liu^3^, Yue Sun^1^, Chunjiang Zhao^3*^, Jihua Tang^2*^, Jinpeng Cheng^2*^

^1^College of Science, Henan Agricultural University, Zhengzhou 450002, China

^2^College of Agronomy, Henan Agricultural University, Zhengzhou 450046, China

^3^National Engineering Research Center for Information Technology in Agriculture, Beijing 100097, China

^*^ Address correspondence to: [zhaocj@nercita.org.cn](mailto:zhaocj@nercita.org.cn) (Z.J.); [tangjihua@henau.edu.cn](mailto:tangjihua@henau.edu.cn) (T.J.); [jpeng_cheng@163.com](mailto:jpeng_cheng@163.com) (C.J.)

**
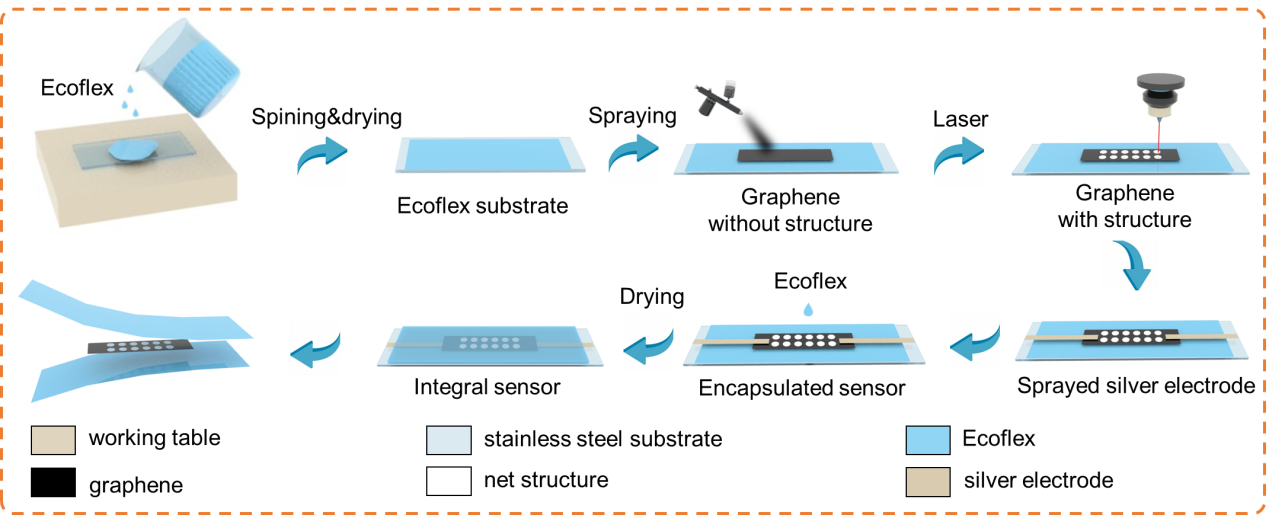
**

**Fig. S1** Fabrication process of the mesh-like stretchable sensors.


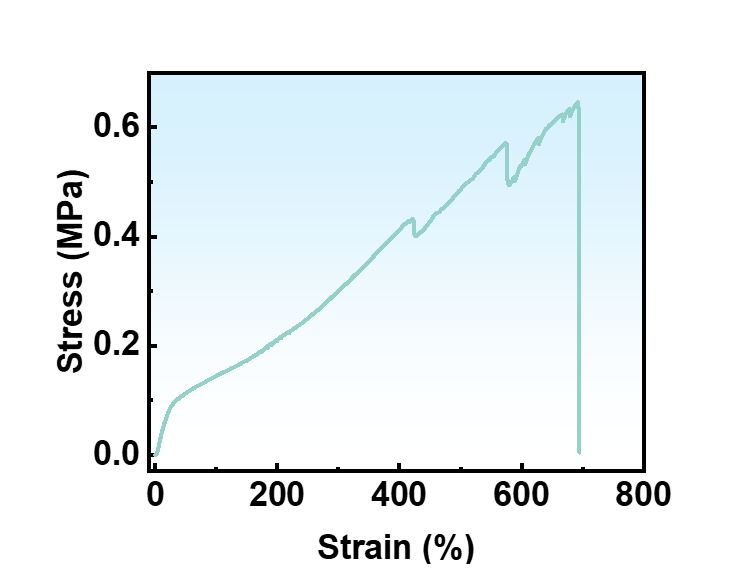


**Fig. S2** Stress-strain curve of the Ecoflex/graphene composite materials.


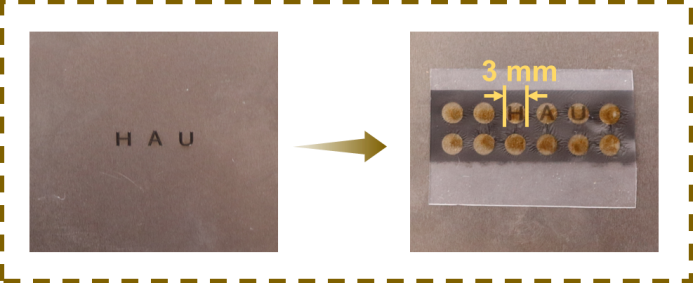


**Fig. S3** Transparency of the Ecoflex/graphene composite materials.


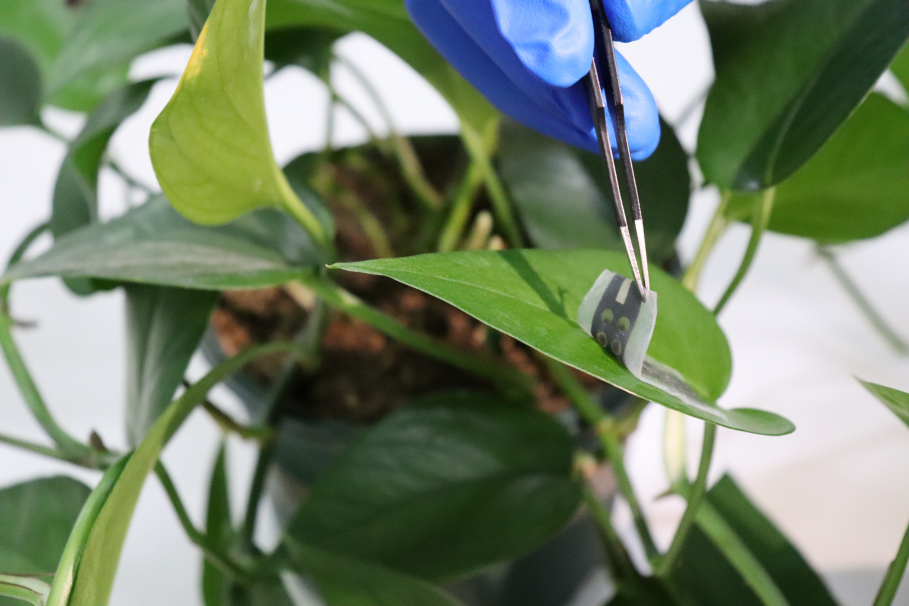


**Fig. S4** Image of sensor physically attached to the plants


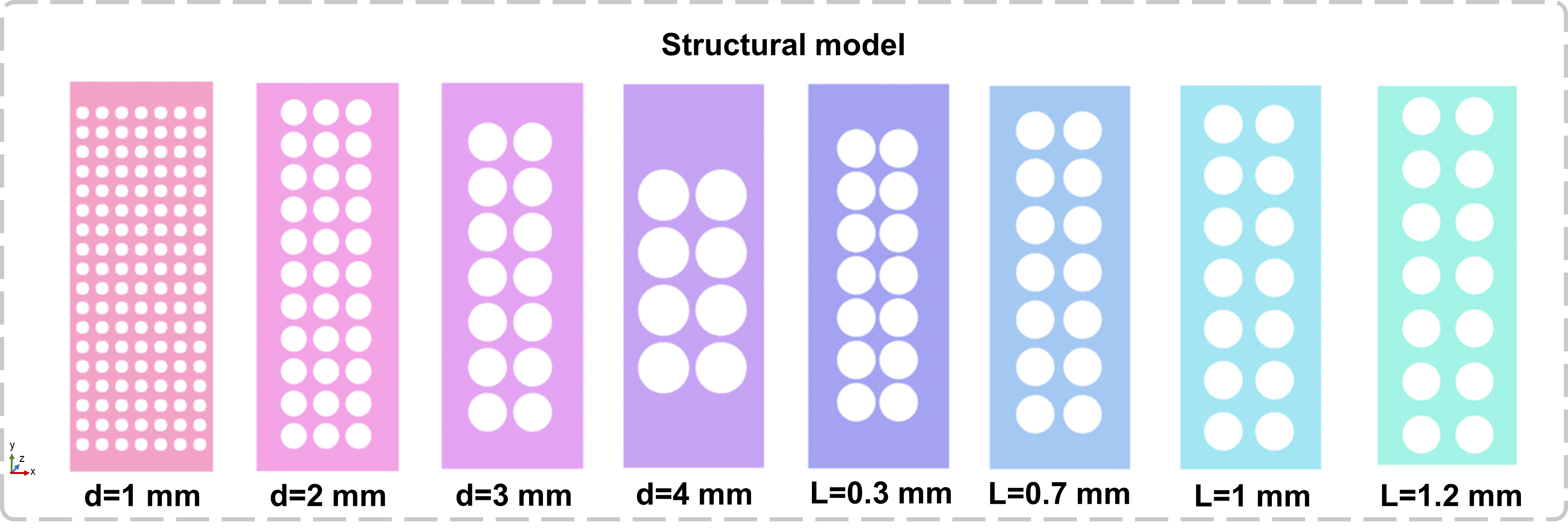


**Fig. S5** Schematic diagram of the mesh-like model obtained through finite element simulation.


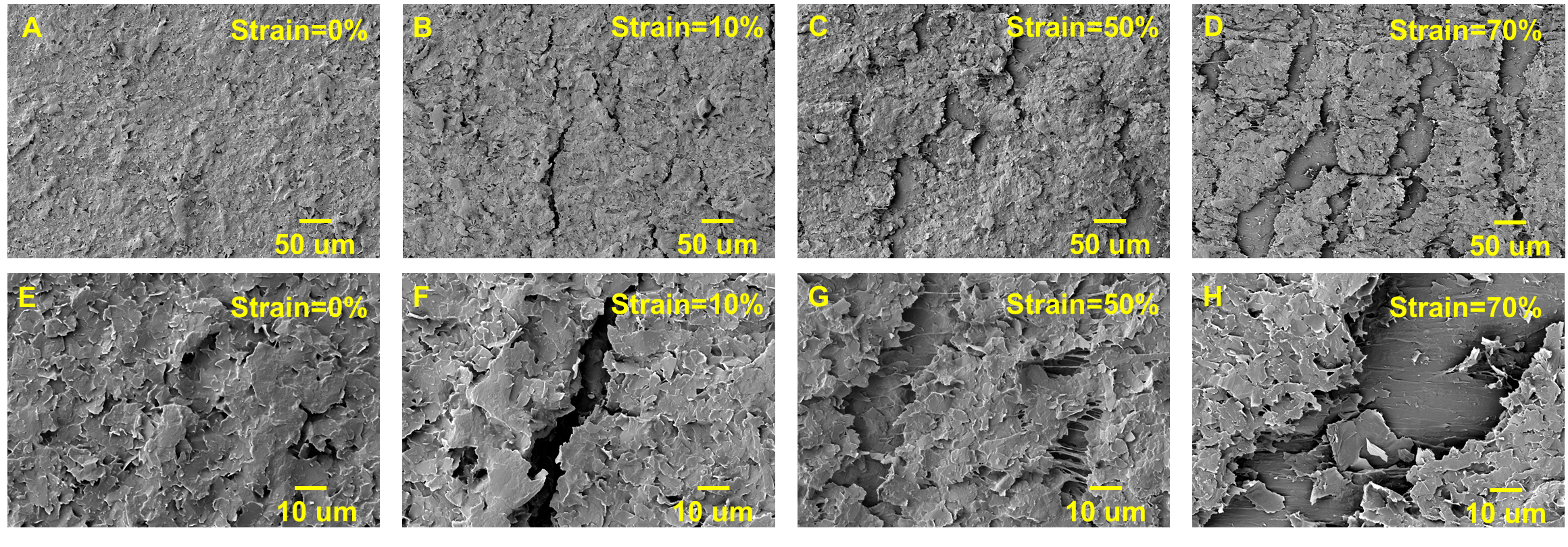


**Fig. S6** (A-D) SEM images of an unencapsulated graphene surface at 0%, 10%, 50%, and 70% strain. (E-H) Magnified morphology SEM images of an unencapsulated graphene surface at 0%, 10%, 50%, and 70% strain.

*
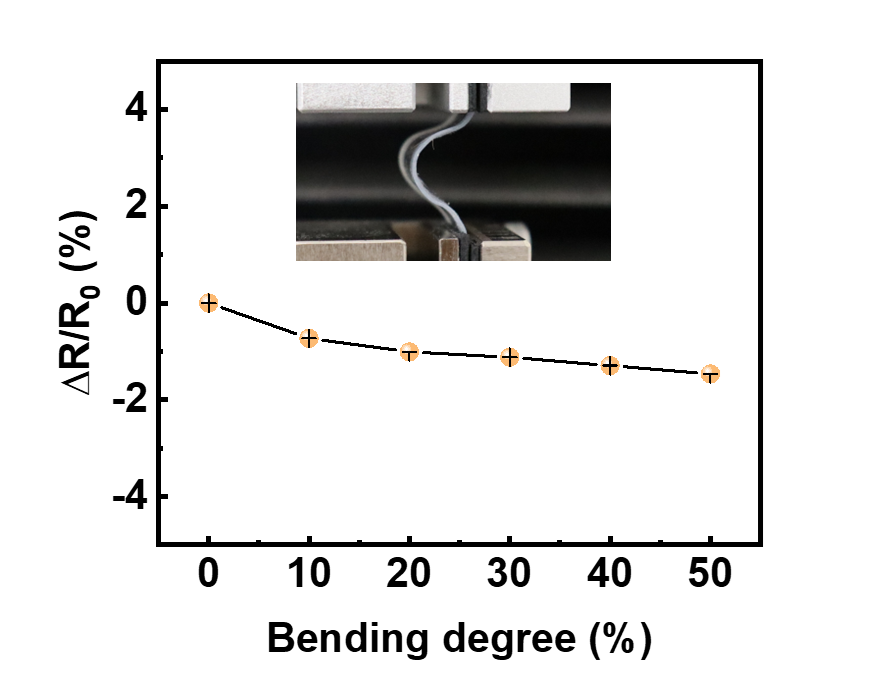
*

**Fig. S7** Relative resistance change rate of sensors under different bending degrees

*
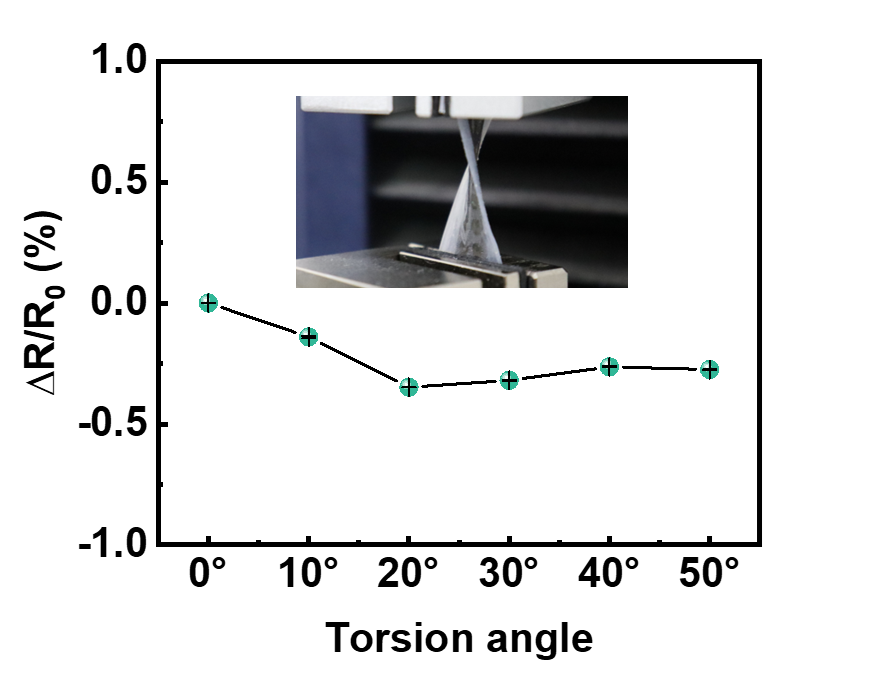
*

**Fig. S8** Relative resistance change rate of sensors under different torsion angles


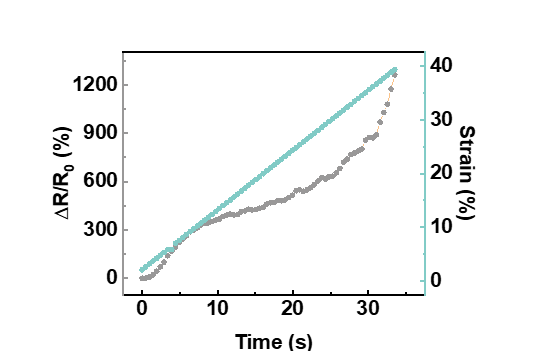


**Fig. S9** Well-synchronized output between resistance-change and tensile strain for the mesh-like stretchable sensor at 40% tensile strain.

**Fig. S10** Stability of the mesh-like stretchable sensors.


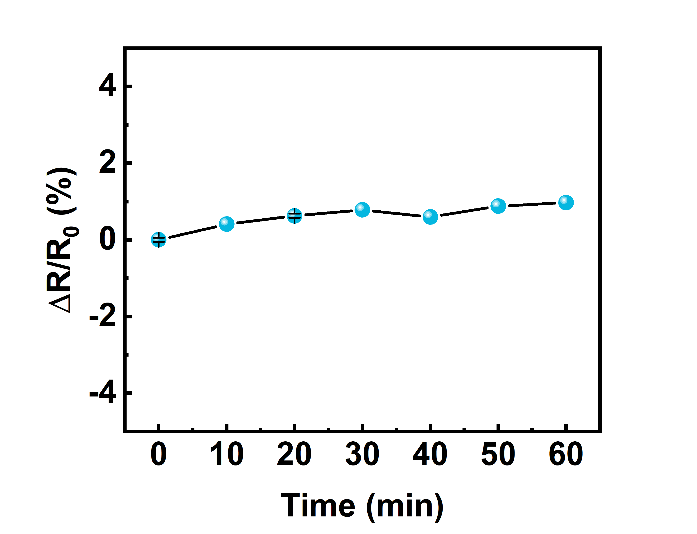


**Fig. S11** Relative resistance change rate of the sensor immersed in an alkaline solution for one hour.

**
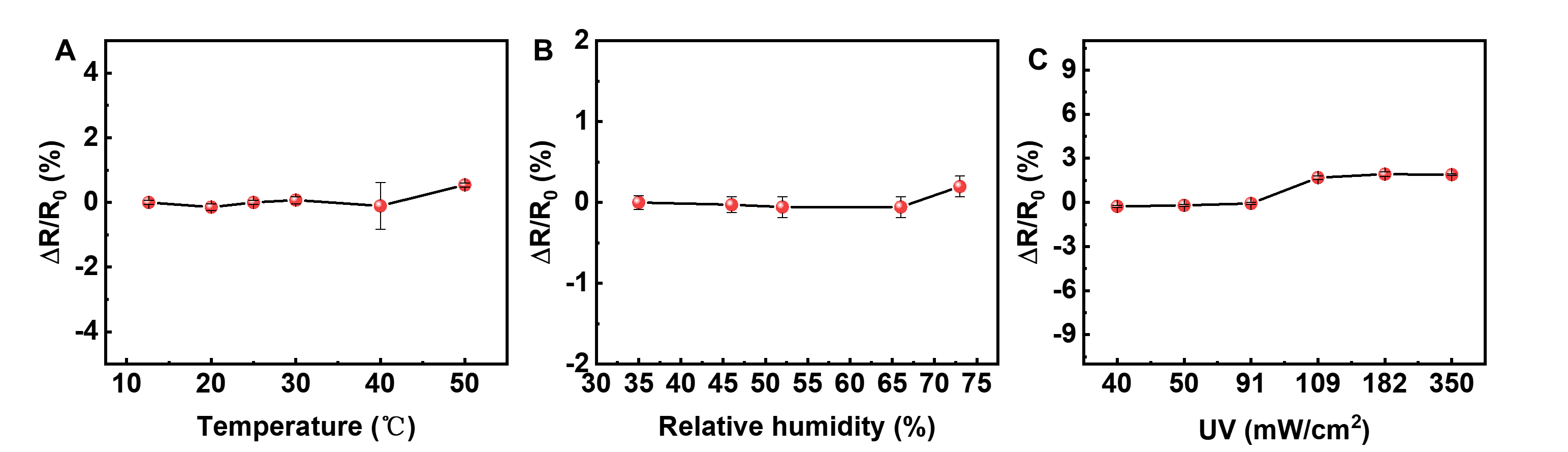
**

**Fig. S12** Relative resistance change rate of sensors under different (A) temperature, (B) humidity, (c) ultraviolet light


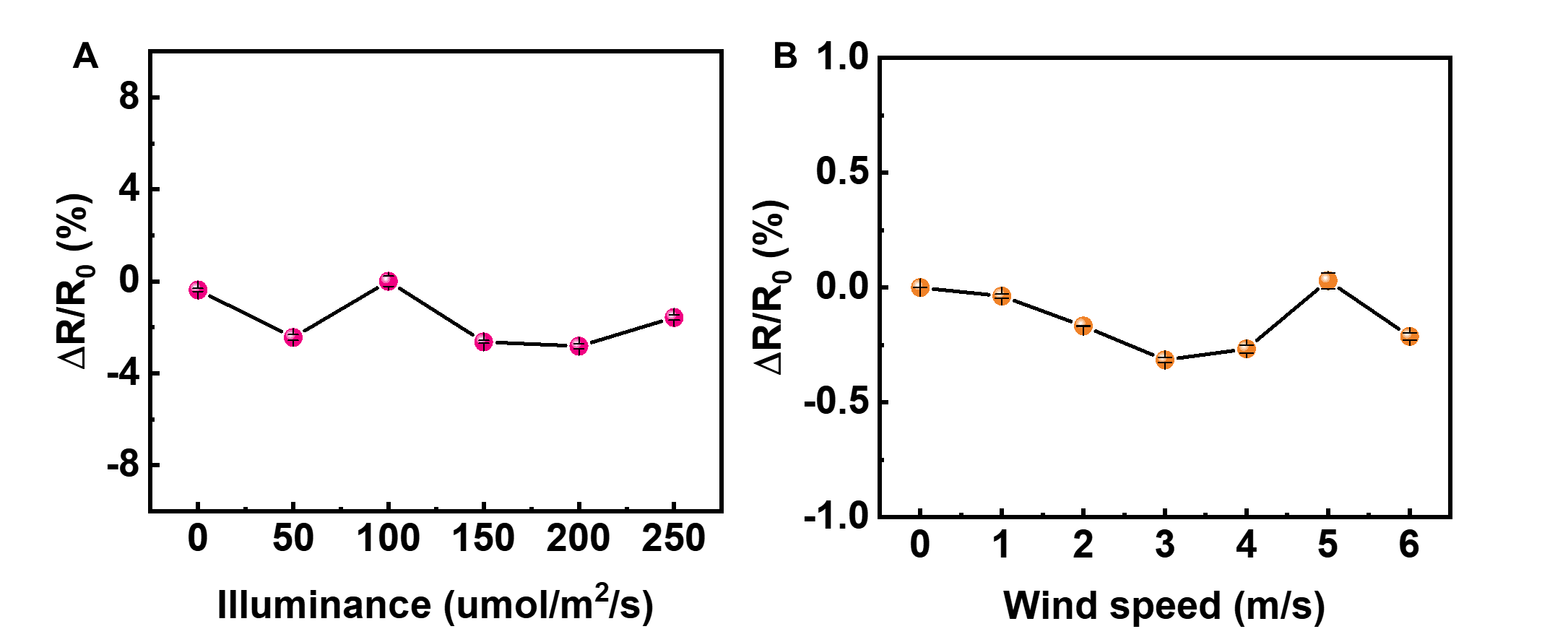


**Fig. S13** Relative resistance change rate of sensors under different (A) illumination, (B) wind speed


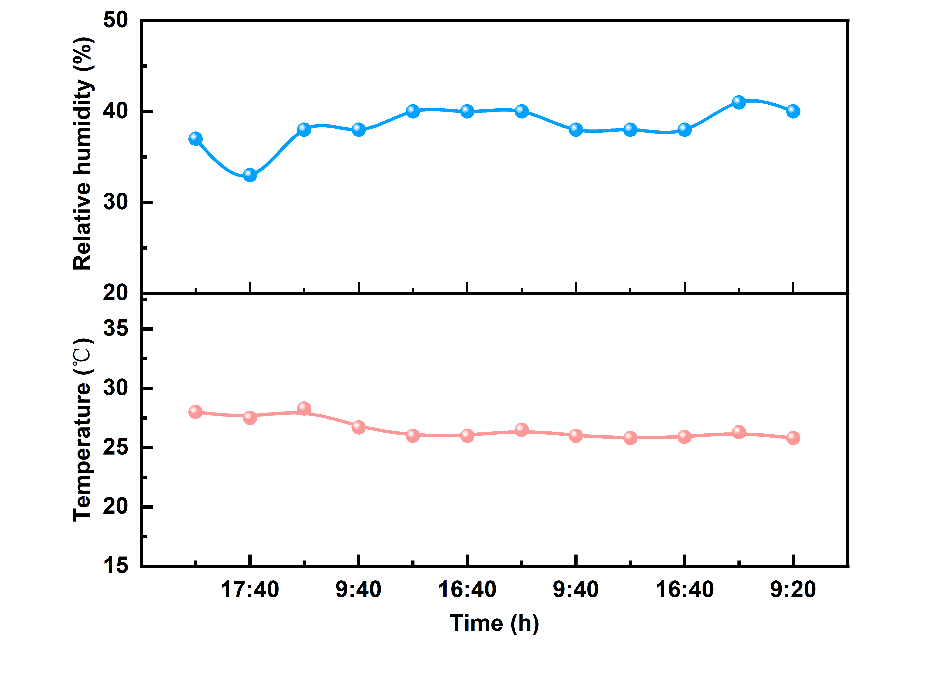


**Fig. S14** The temperature and humidity changes corresponding to the 3-day plant health monitoring


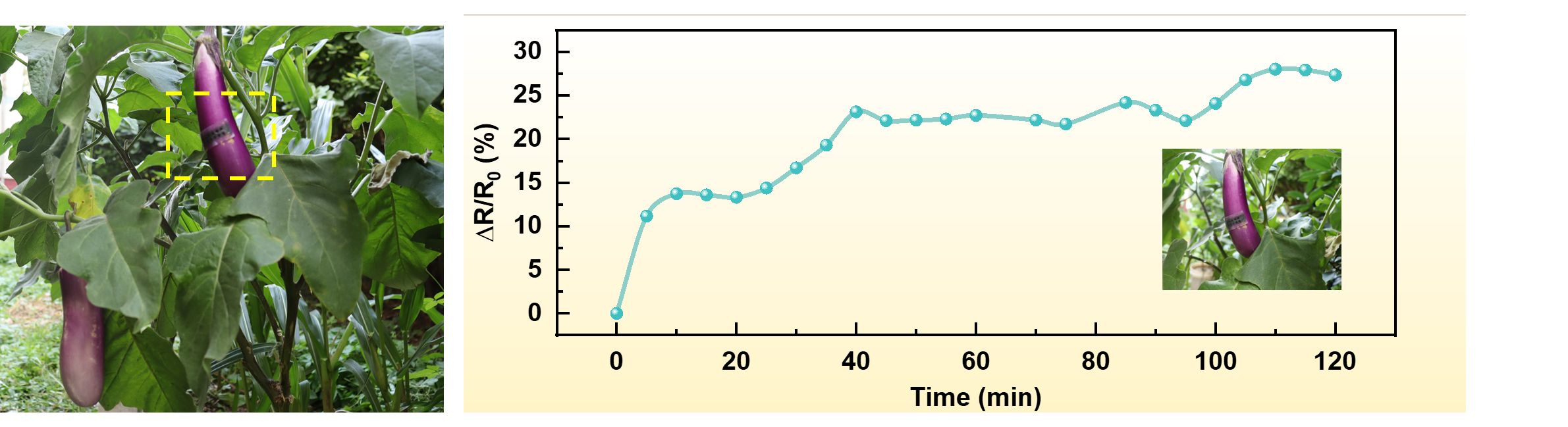


**Fig. S15** The actual deployment of the sensor in an outdoor environment for visualizing the eggplant monitoring for two hours


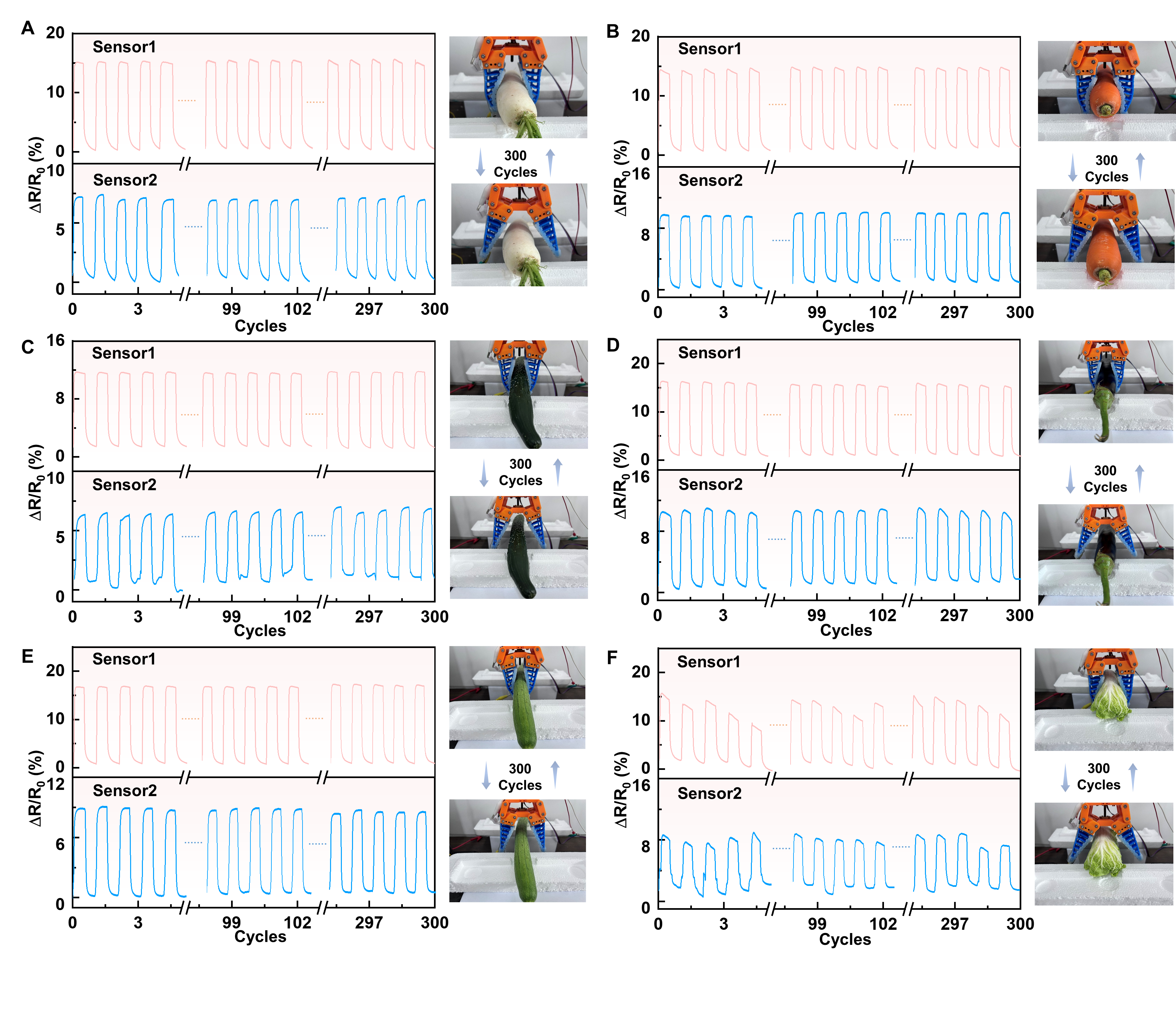


**Fig. S16** Response signals from different types of crops of 300 cycles. (A) White radish. (B) Carrot. (C) Cucumber. (D) Eggplant. (E) *Luffa*. (F) Napa Cabbage.


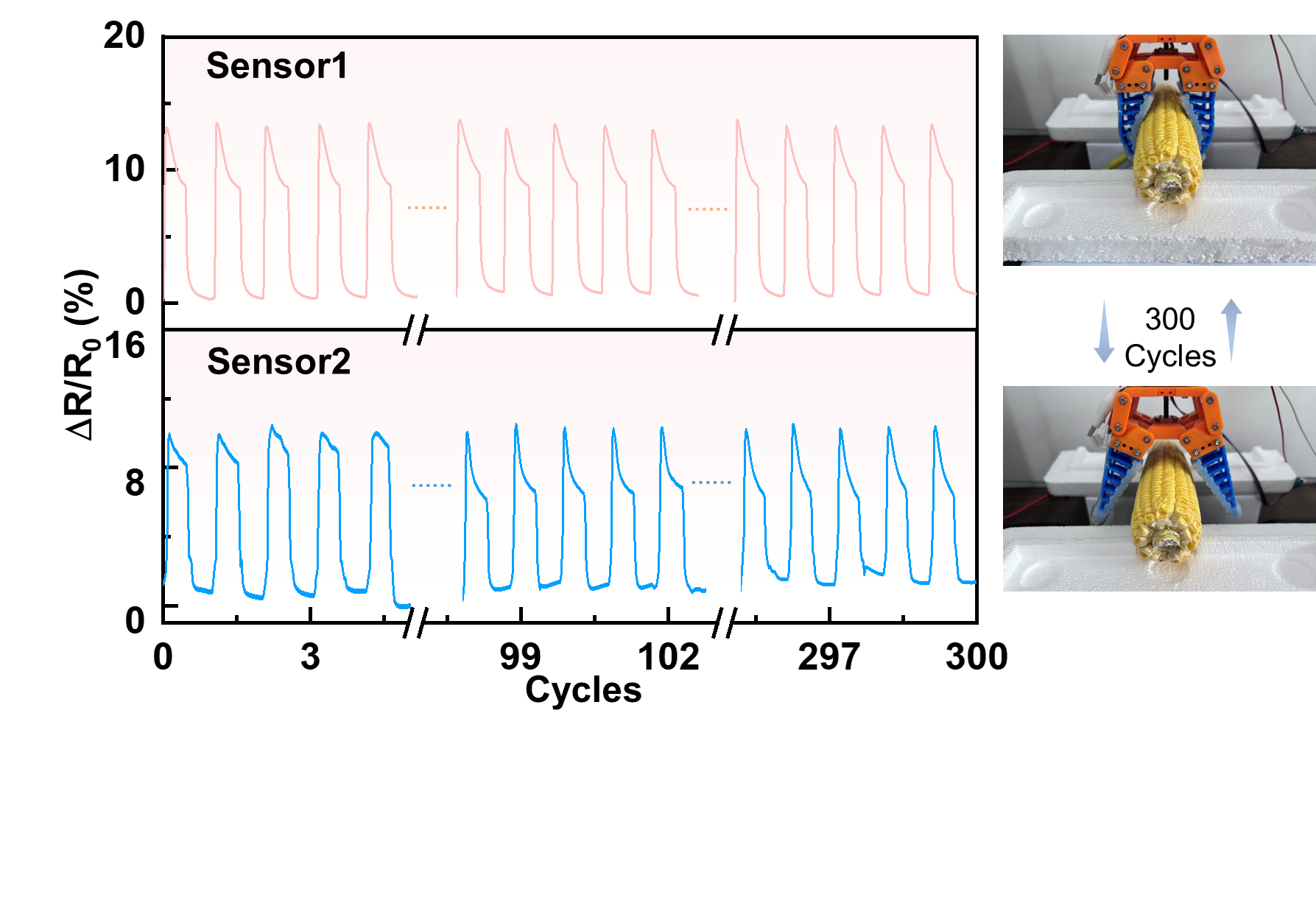


**Fig. S17** Response signals of 300 cycles for maize along with the schematic diagram of the flexible gripper's gripping and releasing actions.


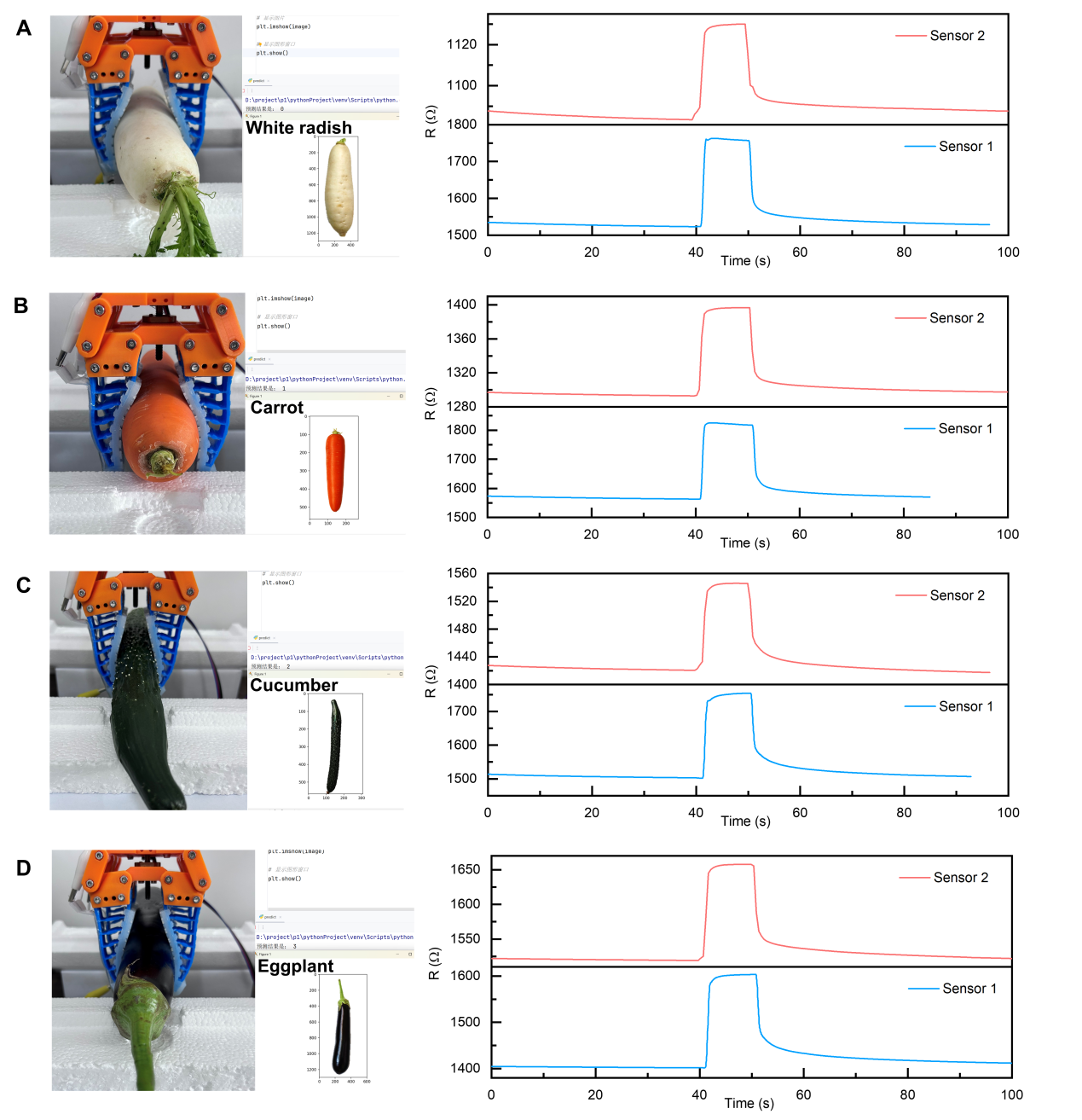


**Fig. S18** One-cycle prediction process and code for different crops along with the corresponding signal response curve. (A) White radish. (B) Carrot. (C) Cucumber. (D) Eggplant.


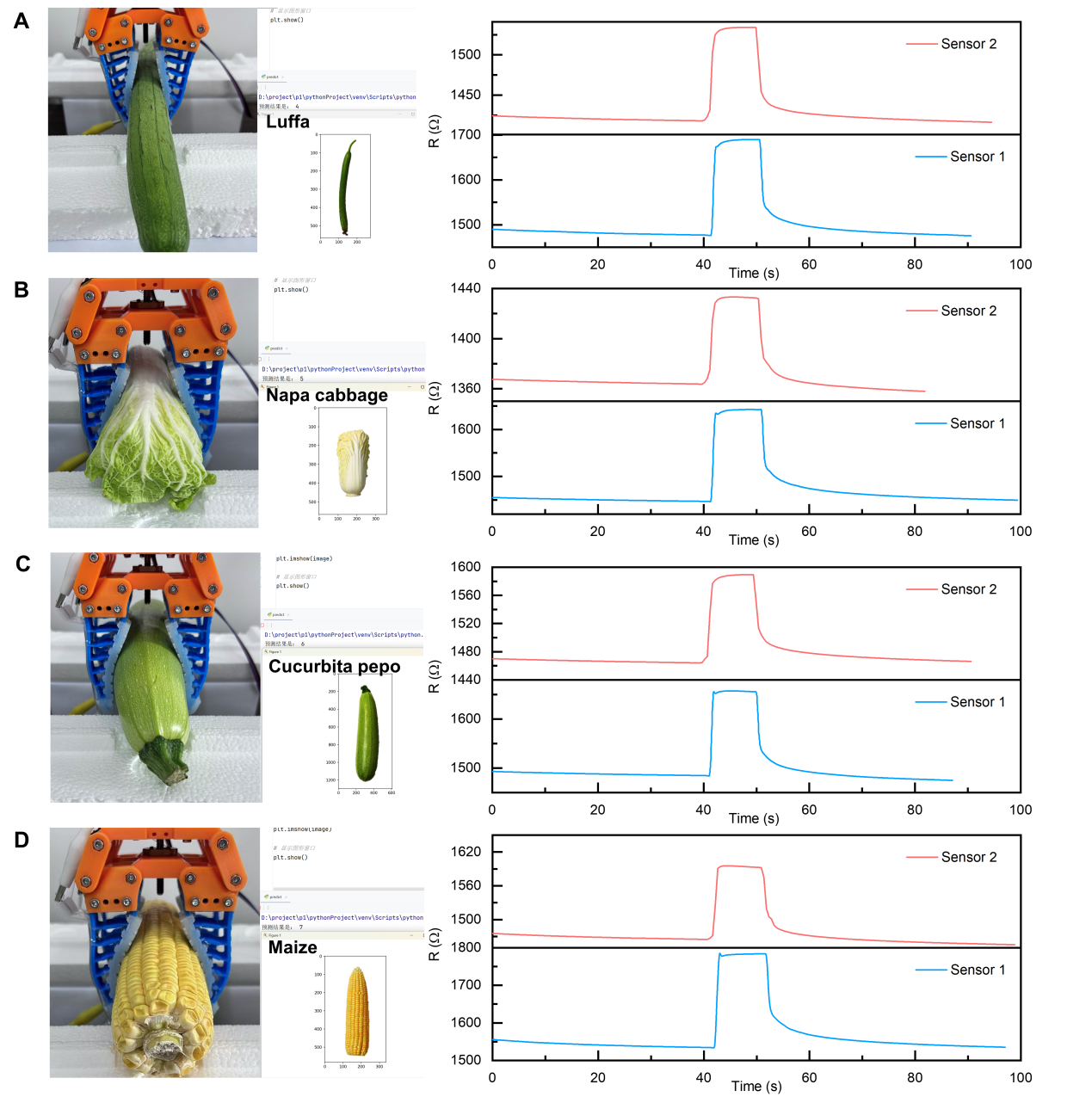


**Fig. S19** One-cycle prediction process and code for different crops along with the corresponding signal response curve. (A) *Luffa*. (B) Napa cabbage. (C) *Cucurbita pepo*. (D) Maize.

**Table S1** Optimization parameters of the circular diameter of the sensor

| Diameter (mm) | Number of circles | Single area  (mm^2^) | Total area of the circle (mm^2^) | Circle area ratio（%） |
| --- | --- | --- | --- | --- |
| 1 | 126 | 0.78 | 98.91 | 30 |
| 2 | 33 | 3.14 | 98.91 | 30 |
| 3 | 14 | 7.06 | 98.91 | 30 |
| 4 | 8 | 12.56 | 98.91 | 30 |

**Table S2** Optimization parameters for the circular spacing of sensors

| Spacing  (mm) | Number of circles | Single area  (mm^2^) | Total area of the circle (mm^2^) | Circle area ratio（%） |
| --- | --- | --- | --- | --- |
| 0.3 | 14 | 7.06 | 98.91 | 30 |
| 0.5 | 14 | 7.06 | 98.91 | 30 |
| 0.7 | 14 | 7.06 | 98.91 | 30 |
| 1.0 | 14 | 7.06 | 98.91 | 30 |

**Movie S1. The ability of the model to accurately identify the eight types of crops.**
